# Molnupiravir and Its Antiviral Activity Against COVID-19

**DOI:** 10.3389/fimmu.2022.855496

**Published:** 2022-04-04

**Authors:** Lili Tian, Zehan Pang, Maochen Li, Fuxing Lou, Xiaoping An, Shaozhou Zhu, Lihua Song, Yigang Tong, Huahao Fan, Junfen Fan

**Affiliations:** ^1^ College of Life Science and Technology, Beijing University of Chemical Technology, Beijing, China; ^2^ Department of Neurology, Institute of Cerebrovascular Disease Research, Xuanwu Hospital, Capital Medical University, Beijing, China

**Keywords:** SARS-CoV-2, COVID-19, molnupiravir, antiviral, orally bioavailable, safety

## Abstract

The coronavirus disease 2019 (COVID-19) pandemic caused by severe acute respiratory syndrome coronavirus 2 (SARS-CoV-2) constitutes a major worldwide public health threat and economic burden. The pandemic is still ongoing and the SARS-CoV-2 variants are still emerging constantly, resulting in an urgent demand for new drugs to treat this disease. Molnupiravir, a biological prodrug of NHC (β-D-N(4)-hydroxycytidine), is a novel nucleoside analogue with a broad-spectrum antiviral activity against SARS-CoV, SARS-CoV-2, Middle East respiratory syndrome coronavirus (MERS-CoV), influenza virus, respiratory syncytial virus (RSV), bovine viral diarrhea virus (BVDV), hepatitis C virus (HCV) and Ebola virus (EBOV). Molnupiravir showed potent therapeutic and prophylactic activity against multiple coronaviruses including SARS-CoV-2, SARS-CoV, and MERS-CoV in animal models. In clinical trials, molnupiravir showed beneficial effects for mild to moderate COVID-19 patients with a favorable safety profile. The oral bioavailability and potent antiviral activity of molnupiravir highlight its potential utility as a therapeutic candidate against COVID-19. This review presents the research progress of molnupiravir starting with its discovery and synthesis, broad-spectrum antiviral effects, and antiviral mechanism. In addition, the preclinical studies, antiviral resistance, clinical trials, safety, and drug tolerability of molnupiravir are also summarized and discussed, aiming to expand our knowledge on molnupiravir and better deal with the COVID-19 epidemic.

## Introduction

The global COVID-19 pandemic is having devastating impacts on human health and economic development. To date, the World Health Organization (WHO) has received reports of more than 412 million confirmed cases of COVID-19, with over 5 million deaths. A total of 10,227,670,521 vaccine doses have been administered by February 13, 2022 (https://covid19.who.int). The common symptoms caused by SARS-CoV-2 infection include fever, cough, fatigue, sore throat, expectoration, muscle ache, headache, pneumonia, dyspnea, and multi-organ damage (1). Vaccines and antiviral drugs are important strategies to prevent and treat COVID-19. A variety of drugs with different mechanisms of action were evaluated in preclinical studies or clinical trials (2, 3). The main drug groups include inhibitors of virus entry into cells (chloroquine, hydroxychloroquine, camostat mesylate, human recombinant soluble ACE2, neutralizing antibodies, nanobodies, miniproteins, convalescent plasma, umifenovir, nitazoxanide, whey protein and lactoferrin) (4–15), inhibitors of SARS-CoV-2 RNA (remdesivir, ribavirin, favipiravir, molnupiravir, and AT-527) (16–20), inhibitors of viral 3CL proteases (lopinavir/ritonavir, PF-07321332, PF-07304814, GC376, and GS-621763) (21–25), inhibitors of host factors essential for virus infection (plitidepsin, fluvoxamine, and ivermectin) (26–28), immunomodulators (dexamethasone, tocilizumab, sarilumab, bevacizumab, eculizumab, and interferons) (29–35). Currently, the U.S. Food and Drug Administration (FDA) has approved remdesivir and several neutralizing antibodies such as bamlanivimab, etesevimab, casirivimab and imdevimab for the treatment of hospitalized COVID-19 patients (36). However, their extensive usage was limited by the complex intravenous administration route and high cost (37, 38). Several SARS-CoV-2 vaccines have been developed and administrated to people worldwide, nevertheless, vaccine breakthrough infections have been widely reported (39–41). Moreover, individuals with immunocompromised may not be fully protected after vaccination, and existing vaccines may not be able to effectively combat the emerging SARS-CoV-2 variants (40, 42, 43). Accordingly, in light of the ongoing pandemic, there is an unprecedently urgent demand for effective treatments to reduce the adverse clinical consequences of SARS-CoV-2 infection.

On October 1, 2021, it was announced that molnupiravir, a drug jointly developed by Merck and Ridgeback Biotherapeutics, can significantly reduce the risk of hospitalization or death in patients with mild or moderate COVID-19, which has attracted widespread attention. The interim analysis of the phase 3 MOVe-OUT clinical trial of a multi-center randomized controlled trial involving 775 subjects showed that 7.3% (28/385) of the patients in the molnupiravir group were hospitalized or died within 29 days, versus 14.1% (53/377) of those in the placebo group, implying that molnupiravir reduced the mortality or hospitalization rate by about 50%. During the 29-day observation period, eight deaths were reported in the placebo group, whereas no patient died in the molnupiravir group. There was no significant difference in any adverse events between the molnupiravir group and the placebo group (35% vs. 40%). Similarly, the incidence of drug-related adverse events was also comparable (12% and 11%, respectively). 3.4% of patients in the placebo group discontinued the study due to adverse events, versus 1.3% of those who received molnupiravir. According to participants with available virus sequencing data (about 40% of participants), molnupiravir showed consistent efficacy among the virus variants Gamma, Delta and Mu. On November 4, 2021, molnupiravir was approved by the U.K. Medicines and Healthcare products Regulatory Agency (MHRA) for the treatment of mild to moderate COVID-19 patients, becoming the world’s first oral anti-COVID-19 drug (https://www.gov.uk/government/news/first-oral-antiviral-for-covid-19-lagevrio-molnupiravir-approved-by-mhra). The all-randomized analysis of the phase 3 study shows a more modest effect of molnupiravir as it decreased the risk of hospitalization or mortality from 9.7% (68/699) in the placebo group to 6.8% (48/709) in the molnupiravir group, for an absolute risk reduction of approximately 3 percentage points (44). On December 23, 2021, the U.S. Food and Drug Administration (FDA) issued an Emergency Use Authorization (EUA) for molnupiravir for the treatment of high-risk adults with mild to moderate COVID-19 (https://www.fda.gov/news-events/press-announcements/coronavirus-covid-19-update-fda-authorizes-additional-oral-antiviral-treatment-covid-19-certain).

**Figure 1:**
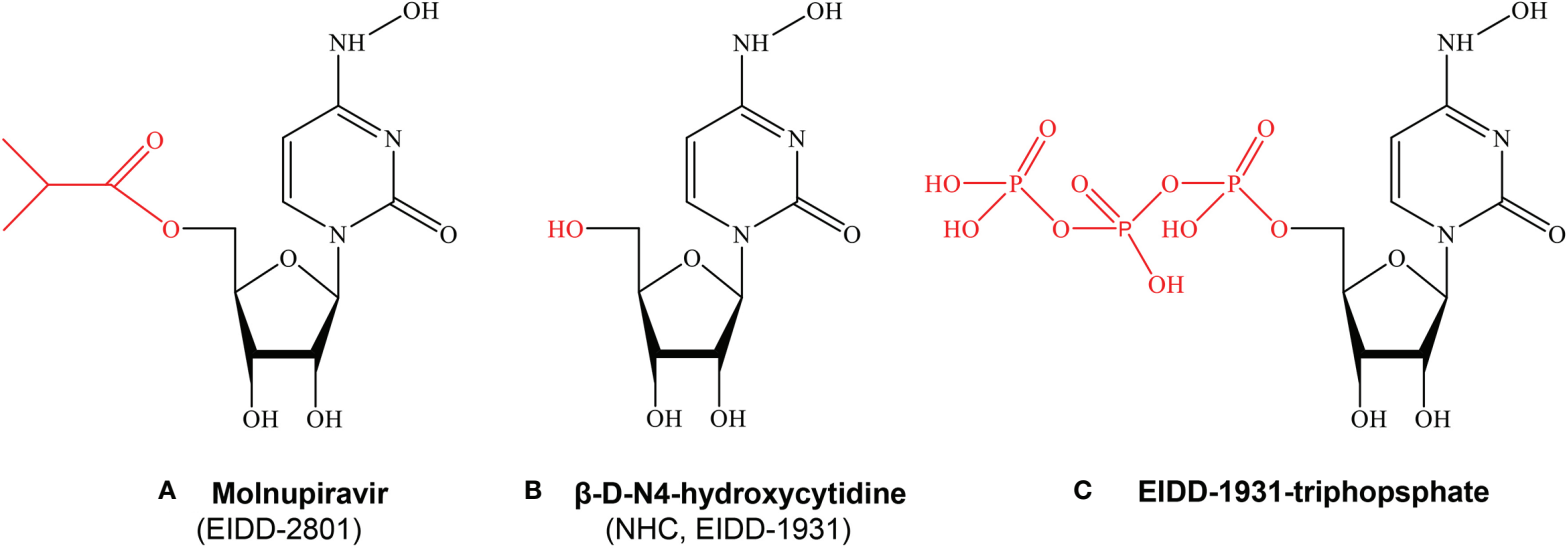
The chemical structures of Molnupiravir, NHC and EIDD-1931-triphopsphate. **(A)** Molnupiravir, Beta-D-N4-hydroxycytidine-5’-isopropyl ester (MK-4482/EIDD-2801) is a bioactive prodrug of NHC (EIDD-1931). **(B)** Beta-D-N4-hydroxycytidine(NHC,EIDD-1931) is an orally bioavailable ribonucleoside analogue, which has broad-spectrum activity against a variety of RNA viruses. **(C)** EIDD-triphosphate can be used as a competitive alternative substrate for viral RNA-dependent RNA polymerase (RdRp), integrates into viral RNA and leads to the accumulation of mutations in the viral genome, resulting in lethal mutations.

**Figure 2:**
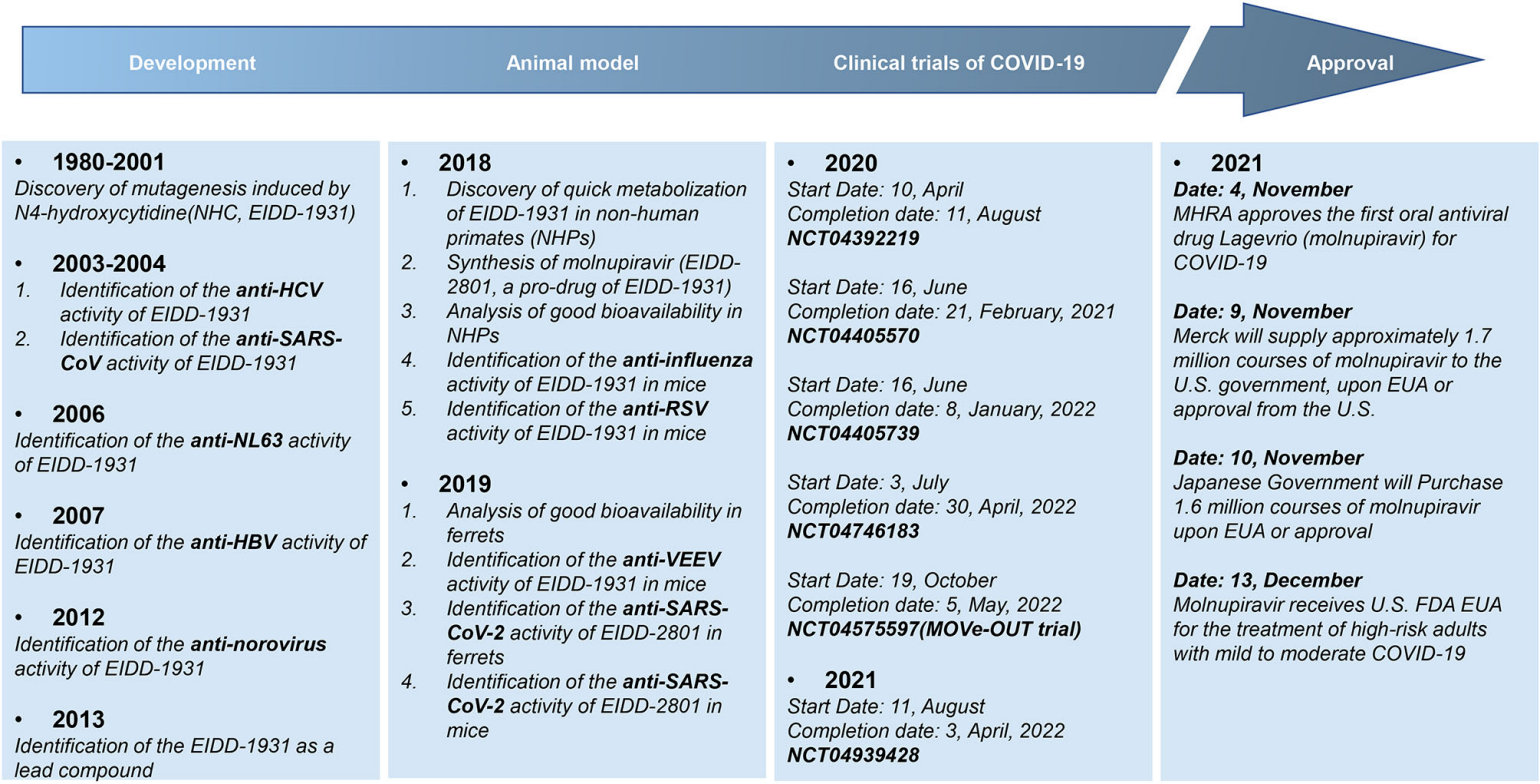
The research history of molnupiravir. Molnupiravir is a prodrug of the ribonucleoside analog β-D-N4-hydroxycytidine (NHC), which has multi-antiviral activities against various viruses including HCV, SARS-CoV, HBV and so on, given the pharmacological mechanism is inducing mutagenesis of viral DNA or RNA.

β-D-N4-hydroxycytidine (NHC, EIDD-1931) is an orally bioavailable ribonucleoside analogue, which has broad-spectrum activity against a variety of RNA viruses (45). Molnupiravir (MK-4482/EIDD-2801), β-D-N4-hydroxycytidine-5’-isopropyl ester, is a bioactive prodrug of NHC (**Figure 1**). Molnupiravir can be administered to patients much more readily than remdesivir or other antiviral biological agents such as convalescent plasma and neutralizing antibodies, which require intravenous or intramuscular injection and hospital use. NHC has been shown to be efficacious against a variety of RNA viruses such as influenza, Ebola virus (EBOV), Venezuelan equine encephalitis virus (VEEV), SARS-CoV-2, SARS-CoV, MERS-CoV, and related zoonotic group 2b or 2c Bat-CoVs in *in vivo* and *in vitro* experiments (46–49). A series of preclinical and clinical trials proved that molnupiravir is safe and effective for the treatment of SARS-CoV-2 infection (50–52). After oral administration, molnupiravir is rapidly transformed into active NHC in plasma, distributed in various organs, and converted into its corresponding 5’-triphosphate by host kinases (45, 53). NHC 5’-triphosphate can be used as a competitive alternative substrate for viral RNA-dependent RNA polymerase (RdRp), integrates into viral RNA and leads to the accumulation of mutations in the viral genome, resulting in lethal mutations (54–56), and the research history of molnupiravir was presented in **Figure 2**.

The potent inhibitory effect of molnupiravir/NHC on a variety of RNA viruses and oral bioavailability highlight its potential utility as an effective drug for SARS-CoV-2 and other future CoVs infection. Therefore, this article will systematically review the latest research progress of molnupiravir from the aspects of its discovery and synthesis, broad-spectrum antiviral effect, antiviral mechanism, preclinical research, antiviral resistance, clinical trials, safety, and drug tolerability, to increase our understanding of molnupiravir about its clinical significance and its possibility for the prevention and treatment of COVID-19.

## Discovery and Synthesis of Molnupiravir

The discovery and early development of molnupiravir began in 2013, aiming to find an oral antiviral drug for the treatment of encephalitic New World alphavirus venezuelan equine encephalitis virus (VEEV) infection (46). Venezuelan equine encephalitis virus as well as eastern and western equine encephalitis virus (EEEV and WEEV) infection can lead to persistent movement disorders and severe neurological sequelae (57). With the increased frequency of their infections, the development of direct-acting antiviral agents became more and more important (58, 59). Researchers at Emory University decided to target the RdRp encoded by RNA viruses and chose to develop ribonucleoside analogue drugs. In 2013, they started screening the activity of ribonucleoside analogues against alphavirus and rapidly determined that N4-hydroxycytidine (identified internally as EIDD-1931) was a precursor with activity against Chikungunya virus (CHIKV) and VEEV (60, 61). Nonetheless, EIDD-1931 was metabolized in the intestinal cells of non-human primates shortly after oral administration. In order to solve this problem, a prodrug of EIDD-1931 (designated EIDD-2801) was synthesized, which promoted the movement of the intestine inner wall and could be effectively delivered to the circulatory system of all tested species including non-human primates (48). Thus, molnupiravir/EIDD-2801 became a development candidate for clinical use. Before the COVID-19 pandemic, molnupiravir was in the preclinical development stage for treating seasonal influenza. With the spread of COVID-19, the development plan has been shifted to highly pathogenic coronavirus infection treatment, and a clinical trial of molnupiravir as an oral therapeutic drug for non-hospitalized COVID-19 patients was conducted (46).

The preliminary study on the synthesis of molnupiravir started with the structural modification of NHC, which is derived from the essential natural product uridine presented in human plasma (62). The synthesis of molnupiravir from uridine is the first-generation synthetic pathway with 5 chemical transformations. But the yield of this synthetic pathway is low, with a maximum of only 17% (63). Alexander Steiner et al. modified this synthesis pathway by simply reordering the original synthesis steps. After the process of triazolation, one-pot acetonide protection/esterification, and telescoped hydroxyamination/continuous flow acetonide deprotection, the yield was increased from 17% to 61%, and fewer separation and purification steps were required (64). Because uridine is an expensive raw material with limited sources, researchers have developed a new strategy to synthesize molnupiravir using cytidine, which not only reduces the cost of raw materials but also simplifies the synthesis to two-step procedures including esterification and transamination. By adjusting the reaction conditions, NHC was synthesized with an analytical yield of 70%. What’s more, pure NHC was obtained by simple crystallization directly from the concentrated reaction mixture, and the isolation yield was 50% (65). This research group then developed a large-scale synthesis method of molnupiravir from cytidine without chromatography, which only required selective enzymatic acylation and transamination, resulting in a total separation yield of 41% (66).

## A Broad-Spectrum of Antiviral Effects

NHC has broad-spectrum antiviral activity against multiple RNA viruses, including bovine viral diarrhea virus (BVDV), hepatitis C virus (HCV), Ebola virus (EBOV), Marburg virus, chikungunya virus (CHIKV), venezuelan equine encephalitis virus (VEEV), influenza virus and respiratory syncytial virus (RSV). NHC was also demonstrated to inhibit human α-CoV HCoV-NL63 and β-CoV SARS-CoV and MERS-CoV. The broad-spectrum antiviral effects of molnupiravir/NHC were summarized in **Table 1**.

**Table 1:** Broad spectrum antiviral effects of molnupiravir/NHC.

Component	Virus	Cell type	Animal	Key findings	Reference
NHC	BVDV	MDBK cells	NA	NHC inhibited cpBVDV RNA production with an EC_90_ of 5.4 ± 0.9 μM. NHC showed no obvious toxicity in confluent MDBK cells (IC_50_>75 μM) but were toxic when cells in exponential growth phase (IC_50 =_ 7 μM). NHC had an EC_90_ of 2 μM in a yield reduction assay.	(67)
NHC	HCV	HCV replicon Huh7 cells	NA	NHC had an EC_90_ of 5 μM on day 4. The HCV RNA reduction was incubation time and nucleoside concentration dependent.	(67)
NHC	EBOV	Vero E6 and macrophages	NA	The EC_50_ of NHC in Vero E6 cells was 3 and 3.8 µM for transcription inhibition and virus spread, respectively. NHC did not have significant effects on cell viability after 48 h incubation and below 12 µM. NHC displayed a moderate cytotoxicity at 24 and 48 µM.	(68)
NHC	MARV	Vero E6 cells	NA	NHC could inhibit MARV replication and spread.	(68)
NHC	CHIKV	Huh7, PBM, Vero, CEM and BHK-21 cells	NA	NHC has little or no effect on CHIKV entry but inhibit CHIKV early-stage replication with an EC_50_ of 0.8 μM in the Huh-7–CHIKV replicon cell line and 0.2 μM in Vero cells. The CC_50_ for NHC were 30.6 μM, 7.7 μM, and 2.5 μM in PBM, Vero, and CEM cells, respectively.	(60)
NHC	VEEV	Vero cells	NA	NHC is a very potent anti-VEEV agent and the EC_50_, EC_90_, and EC_99_ were 0.426, 1.036, and 2.5 μM, respectively, as well as CC_50_>200 μM. The antiviral effect of NHC is more prominent when applied earlier post-infection. NHC induced high mutation rates in VEEV G RNA. VEEV resistance to NHC developed very inefficiently.	(61)
EIDD-1931/NHC	VEEV	NA	ICR mice	NHC is orally available and quickly distributed to brain tissue and converted to active 5’-triphosphate form. NHC has prophylactic and protective effects against VEEV infection in mice.	(69)
NHC	RSV, IAV	HEp-2 cells, HBTECs, MDCK cells	BALB/cJ mice, guinea pigs	NHC showed potent antiviral activity against different IAVs, IBVs, and RSVs both *in vitro* and *in vivo*. It suppressed IAV spread to uninfected contact animals.	(48)
NHC	SARS-CoV	Vero 76 cells	NA	CPE reduction and NR assays demonstrated that NHC inhibits SARS-CoV with an EC_50_ of 5μM and IC_50_ of 50 μM. NHC reduced SARS-CoV yields with an EC_90_ of 6 μM by virus yield reduction assay.	(70)
NHC	HCoV-NL63	LLC-MK2 cells	NA	NHC inhibits HCoV-NL63 replication, the IC_50_ value is about 400 nM, and the CC_50_ value is about 80 μM.	(71)
NHC/EIDD-1931	MHV, MERS-CoV	Vero cells, DBT-9 cells	NA	NHC significantly inhibited MHV (EC_50 _= 0.17 μM) and MERS-CoV (EC_50 _= 0.56 μM) in a dose-dependent manner with low cytotoxicity. The NHC inhibition profile of MHV is consistent with mutagenesis and poses a high genetic barrier to resistance for β-CoVs.	(55)

NHC, β-D-N(4)-hydroxycytidine; BVDV, bovine viral diarrhea virus; MDBK, Madin-Darby bovine kidney; NA, Not Available; EC_90_, 90% effective concentration; IC_50_, 50% inhibitory concentration; HCV, hepatitis C virus; EBOV, Ebola virus; CC_50_, 50% cytotoxicity concentration; MARV, Marburg virus; CHIKV, Chikungunya virus; PBM, peripheral blood mononuclear; BHK-21, baby hamster kidney cells; EC_50_, 50% cytotoxicity concentration; VEEV, Venezuelan equine encephalitis virus; EC_99_, 99% effective concentration; RSV, respiratory syncytial virus; IAV, influenza A virus; IBVs, influenza B virus; HBTECs, human bronchial tracheal epithelial cells; MDCK, Madin-Darby canine kidney; SARS CoV, Severe acute respiratory syndrome; HCoV-NL63, Human coronavirus NL63; MHV, murine hepatitis virus; MERS-CoV, Middle East respiratory syndrome CoV; DBT, Murine astrocytoma delayed brain tumor.

NHC was found to inhibit bovine viral diarrhea virus and hepatitis C virus in a dose-dependent manner. In 3H-labeled NHC treated HCV replicon cells, NHC can be quickly converted into the monophosphate, diphosphate and triphosphate (MP, DP and TP). The highest content of NHC-TP was detected, and it is expected that NHC-TP to be the primary molecular form with antiviral activity in the replication system. In cell-free HCV NS5B polymerization reactions, NHC-TP did not show an inhibitory effect on the viral polymerase, but NHC can interfere with the cytoplasmic RNA metabolism of HCV- infected cells (67). Moreover, NHC was certified to inhibit the replication and spread of Ebola virus and Marburg virus *in vitro*. The flow cytometry results indicated that without NHC treatment, about 20% of the cells were infected (GFP positive) on day 3 after Ebola virus infection, whereas 1.6% of those in the presence of NHC. Median fluorescence intensity (MFI) analysis showed that NHC dramatically decreased Ebola virus transcription in the first 2 days post-infection. However, about 18% of the cells in the NHC group showed GFP expression 4 days after infection, suggesting that NHC treatment led to the delay rather than complete blocking of Ebola virus replication (68). Utilizing a small-molecule library containing more than 600 compounds, Ehteshami et al. demonstrated that NHC is a novel inhibitor of chikungunya virus (CHIKV). The antiviral activity, toxicity, intracellular metabolism, and mechanism of action of NHC on chikungunya virus were systematically investigated. The results showed that NHC had little effect on chikungunya virus entry, but inhibited chikungunya virus replicon activity in Huh-7–CHIKV and BHK-21 replicon cell lines as well as impaired infectious chikungunya virus replication in Vero cells. NHC inhibited chikungunya virus with a concentration for 50% of maximal effect (EC_50_) value of 0.8 μM in the Huh-7–CHIKV replicon cell line and 0.2 μM in Vero cells. The 50% cytotoxicity concentration (CC_50_) values for NHC were 30.6 μM, 7.7 μM and 2.5 μM in peripheral blood mononuclear (PBM), Vero and CEM cells, respectively. Liquid chromatography-tandem mass spectroscopy (LC-MS/MS) showed that NHC-TP is the most abundant metabolite. It was speculated that NHC-TP can directly target viral polymerase and act as a non-specific chain terminator. Alternatively, the incorporation of NHC-TP into viral RNA may increase the mutation rate, and its exact mechanism remains to be determined (60). Moreover, NHC can effectively inhibit venezuelan equine encephalitis virus (VEEV), and the concentration for 50% of maximal effect (EC_50_) is 0.426 μM. NHC treatment resulted in a moderate reduction of viral particle release and a significant reduction of particle infectivity. This antiviral activity is determined by the accumulation of virus-specific RNA mutations induced by NHC. NHC is most effective against VEEV when applied in the early stage of infection, implying the importance of early medication after virus infection. In addition, this study found that NHC has a high genetic barrier to VEEV resistance, as low-level resistance requires multiple mutations in VEEV specific RdRp NSP4 gene (61). Another study found that after oral administration, NHC/EIDD-1931 level increased rapidly in mouse plasma, quickly distributed to organs including brain, and converted into active 5’-triphosphate form. EIDD-1931 was shown to have prophylactic and protective effects against VEEV infection in mice. Prophylactic efficacy study proved that EIDD-1931 reduced the mortality rate by 80%-90%. The therapeutic study found that EIDD-1931 is most effective when it is applied early after VEEV infection, as all animals survived when treatment initiated at 6 and 12 h after virus challenge, compared with 90% of those survived when treatment started 24 h post-infection and 40% of mice survived when treatment delayed at 48 h post-challenge (69). Therefore, NHC is a promisingly effective anti-VEEV drug and may also be used to treat other alphavirus infections. Using the dual pathogen high-throughput screening of influenza A virus (IAV) and respiratory syncytial virus (RSV) inhibitors, the researchers determined that NHC is a potent inhibitor of RSV, influenza B virus (IBV) and influenza virus (IAV) from human, avian, and swine. NHC did not show robust resistance to RSV-A2 and IAV-WSN *in vitro*. And it exhibited high metabolic stability in primary human bronchial tracheal epithelial cells (HBTECs) and good oral bioavailability and tolerability in mice. This compound could reduce the viral load in the lung and alleviate the symptoms in RSV, seasonal influenza viruses and highly pathogenic avian influenza virus (IAV) infected mouse model. Furthermore, it can block virus spread in guinea pig IAV transmission model. These studies demonstrated that NHC is a therapeutic candidate for influenza-like diseases (48).

NHC was also found to inhibit coronavirus. Barnard et al. found that NHC was one of the active compounds against SARS-CoV with a 90% effective concentration (EC_90_) of 6 μM (70). NHC also inhibits HCoV-NL63 replication, the 50% inhibitory concentration (IC_50_) value is about 400 nM, and the 50% cytotoxicity concentration (CC_50_) value is about 80 μM (71). NHC has been proved to inhibit murine hepatitis virus (MHV) (EC_50 _= 0.17 μM) and MERS-CoV (EC_50 _= 0.56 μM) in a dose-dependent manner with low cytotoxicity. Time of drug addition assays showed that NHC significantly inhibited MHV replication at early stages of the viral replication cycle. The types of mutations induced by NHC were augmented with the raise of NHC concentration, and NHC increased the number and ratio of G:A and C:U transition mutations after a single infection. It was found that NHC inhibition is modestly affected by ExoN-mediated proofreading activity (55).

## Mechanism of Action

Although molnupiravir and remdesivir are both nucleoside analogues, significant differences exist in their action mechanisms. Several studies have clarified the mechanisms of molnupiravir, highlighting its important role in suppressing SARS-CoV-2 infection (49, 56, 72).

**Figure 3:**
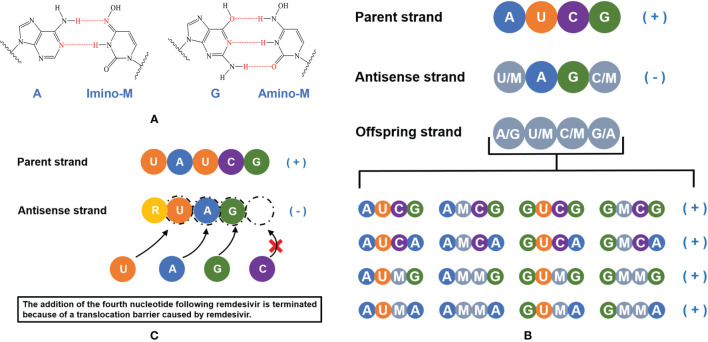
A schematic illustration of the mechanism of molnupiravir and remdesivir. **(A)** NHC monophosphate (M) can bind to A or G. **(B)** NHC triphosphate can be used as a substrate for RNA synthesis, the addition of NHC monophosphate (M) does not terminate the extension of anti-sense RNA chain but induces accumulating mutations. **(C)** The active form of remdesivir acts as a nucleoside analog, remdesivir triphosphate (RTP) can be used as a substrate and the remdesivir monophosphate (R) can pair with uridine monophosphate (U) in the RNA template strand. The addition of the fourth nucleotide following remdesivir is terminated because of a translocation barrier, thus the RNA replication will be impacted.

In order to clarify the antiviral mechanism of molnupiravir, researchers have conducted several experiments. It was demonstrated that NHC can participate in viral replication and increase G to A and C to U substitution, introducing mutations in viral synthesis and inactivating the progeny viruses (72). Normally, G pairs with C (G:C) and A pairs with U or T (A:U/A:T) according to the principle of complementary base pairing, but NHC can pair with A or G, respectively. After incorporated into the template strand, the extension of NHC:A or NHC:G product can be mildly inhibited by U:A or C:G, but this inhibition can be weakened when the concentration of NHC is sufficiently high (72). In subsequent replication, mutations from G to A (G:NHC:A) and C to U (C:G:NHC:A:U) could be observed as NHC pairs with A or G, which is probably related to the strong antiviral function of molnupiravir (72, 73). In addition, Cramer P and colleagues quantitatively studied the effect of NHC on RNA synthesis (56). They demonstrated that NHC monophosphate (M) can bind to A or G with two different tautomeric forms by RNA extension analysis, thus NHC can be used as a substrate for RNA synthesis (**Figure 3A**). And the insertion of M would not affect the extension of RNA strand, which allows the strand containing M can be synthesized with the existence of RdRp and NHC triphosphate (MTP). However, when the anti-sense RNA strand containing M nucleotides used as a template strand, the corresponding positions of M, A, G are paired with A/G, U/M, C/M randomly in the offspring RNA strand, causing mutations in the offspring strand (**Figure 3B**). With the accumulation of mutations, normal viral proteins could not be translated from offspring strands, which are eventually lethal to virus.

Remdesivir, another nucleoside analog, is the first approved antiviral treatment against COVID-19 (https://www.ema.europa.eu/en/human-regulatory/overview/public-health-threats/coronavirus-disease-covid-19/treatments-vaccines-covid-19#remdesivir-section). Compared with molnupiravir, remdesivir would not cause mutations of RNA. After remdesivir incorporates into the RNA strand, the addition of the fourth nucleotide will be terminated because of a translocation barrier (**Figure 3C**). However, the translocation barrier caused by remdesivir could be eliminated at a high NTP concentration system, which may partly explain why the anti-SARS-CoV-2 activity of remdesivir in clinical trials is not satisfactory (74). In addition, the M:G and M:A base pairs formed by NHC monophosphate are stable, which avoid M being eliminated by Nsp14 exonucleases, this may be the reason why molnupiravir has higher antiviral activity than remdesivir. *In vivo* and *in vitro* experiments showed that the decreased MERS-CoV viral load was related to the increased mutation frequency (49), which also supported the conclusion of Gordon et al. and Kabinger et al. (56, 72).

The above studies analyzed the mechanism of molnupiravir and showed that it can be used as an effective antiviral drug to combat COVID-19 and other zoonotic diseases.

## Preclinical Studies

As an orally bioavailable nucleoside analogue prodrug of NHC/EIDD-1931, molnupiravir/EIDD-2801 has been subjected to a series of preclinical experiments. In contrast to the prototype drug EIDD-1931, EIDD-2801 has better oral bioavailability in mice, nonhuman primates and ferrets, which can be efficiently hydrolyzed *in vivo* and has similar drug action level (49, 50). Therefore, EIDD-2801 is commonly used for *in vivo* efficacy testing, while NHC is used for cell culture experiments. The effectiveness and safety of EIDD-2801 have been demonstrated from several aspects, which lay the foundation for further clinical use.

## *In Vitro* Efficacy of Molnupiravir

Studies in primary human cells and other cell lines have certified that NHC has potent antiviral activity against a variety of coronaviruses such as SARS-CoV, SARS-CoV-2 and MERS-CoV. The 50% inhibitory concentration (IC_50_) value of molnupiravir against SARS-CoV-2 was 0.3 µM in Vero cells and 0.08 µM in human lung epithelial cell line Calu-3 (49). The 50% effective concentration (EC_50_) value of NHC against SARS-CoV-2 virus-infected Vero E6-GFP and Huh7 cells was 0.3 µM and 0.4 µM, respectively (75). Using human tracheal airway epithelial cells (HtAEC) and human small airway epithelial cells (HsAEC) for screening drugs against SARS-CoV-2, Do et al. found that EIDD-1931 could dose-dependently inhibit viral RNA replication and reduce infectious virus titers in the supernatant (76). By developing a SARS-CoV-2 RdRp-dependent Gluc reporter system, Zhao et al. demonstrated that molnupiravir strongly inhibited SARS-CoV-2 RdRp and the EC_50_ was 0.22 μM (77). Cox  et al. validated the potency of NHC against the clinical isolate of SARS-CoV-2 in cell culture and revealed that the 50% and 90% effective concentration (EC_50_ and EC_90_) values were 3.4 µM and 5.4 µM, respectively (78). The above results verified the effectiveness and low cytotoxicity of NHC against SARS-CoV-2 in different cell lines, highlighting its significance for clinical research (**Table 2**).

**Table 2:** *In vitro* anti-SARA-CoV-2 activity of molnupiravir/NHC in cell-based assays.

Component	Virus	Cell line	Key results	Reference
NHC	SARS-CoV, SARS-CoV-2 and MERS-CoV	primary human airway epithelial (HAE) cells	NHC is active against several coronaviruses including SARS-CoV, SARS-CoV-2 and MERS-CoV. The IC_50_ of molnupiravir against SARS-CoV-2 was 0.3 µM in Vero cells and 0.08 µM in human lung epithelial cell line Calu-3.	(49)
NHC	SARS-CoV-2	Vero E6-GFP and Huh7 cells	The EC_50_ value of NHC against SARS-CoV-2 virus-infected Vero E6-GFP and Huh7 cells was 0.3 µM and 0.4 µM, respectively.	(75)
NHC/EIDD-1931	SARS-CoV-2	Human tracheal airway epithelial cells (HtAECs) and human small airway epithelial cells (HsAECs)	EIDD-1931 could dose-dependently inhibit viral RNA replication and reduce infectious virus titers in the supernatant.	(76)
Molnupiravir	SARS-CoV-2	HEK-293 cells	Molnupiravir showed strong inhibition of SARS-CoV-2 RdRp activity with an EC50 value of 0.22 μM.	(77)
NHC	2019-nCoV/ USA-WA1/2020	Vero E6 cells	Four-parameter variable slope regression modeling of the dose-response data revealed that the EC_50_ and EC_90_ value were approximately 3.4 µM and 5.4 µM, respectively.	(78)

## Efficacy in Animal Models

Animal models can be used to investigate the pathogenicity and transmissibility of emergent highly pathogenic human coronaviruses and to evaluate the *in vivo* inhibitory activity of novel drugs. Molnupiravir has been shown to display potent broad-spectrum antiviral activity in murine, syrian hamster and ferret models, which provide strong evidence of the promising role of this compound for human clinical antiviral therapy (**Table 3**).

**Table 3:** *In vivo* anti-SARS-CoV-2 activity of molnupiravir/EIDD-2801 in animal studies.

Component	Virus	Animal model	Key findings	Reference
Molnupiravir/EIDD-2801	SARS-CoV-2	cynomolgus monkey and ferret model	Studies showed that EIDD-2801 achieves efficient biodistribution and anabolism *in vivo* at the orally administered concentration of 128 mg/kg and a single oral dose of EIDD-2801 at 128mg/kg in ferrets resulted in EIDD-1931 lung concentrations of 10.7 ± 1.2 nmol/g.	(79)
Molnupiravir/EIDD-2801	SARS-CoV-2	immunodeficient mice implanted with human lung tissue (LoM model)	Therapeutic results indicated that molnupiravir/EIDD-2801 administration greatly reduced the number of infectious particles in LoM human lung tissue by more than 25,000 times (P=0.0002). When treatment was initiated 48 h post-exposure, the virus titer was significantly reduced by 96% (P=0.0019).	(50)
Molnupiravir/EIDD-2801	SARS-CoV-2	C57BL/6 mouse model	Prophylactic or therapeutic administration of EIDD-2801 significantly diminished body weight loss, greatly reduced lung hemorrhage and decreased virus titer in the lungs in a dose-dependent manner after SARS-CoV-2 infection.	(49)
Molnupiravir/EIDD-2801	SARS-CoV-2	Syrian hamster	EIDD-2801 could significantly reduce viral RNA genome copy number and infectious virus levels.	(80)
Molnupiravir	Wuhan strain B.1.1.7 and B.1.351 variants	Syrian hamster	Molnupiravir treatment significantly reduced viral RNA copy number and infectious virus titers in lung regardless of the SARS-CoV-2 variants.	(19)
Molnupiravir/EIDD-2801	SARS-CoV-2	ferret model	EIDD-2801 could dramatically decrease SARS-CoV-2 load in the upper respiratory tract and thoroughly block virus spread in ferrets. Molnupiravir treatment did not cause any obvious adverse effects.	(78)
Molnupiravir	SARS-CoV-2	ferret model	Suboptimal doses of favipiravir or molnupiravir lead to approximately 1.2 log10 reduction in lung infectious virus titers, the combination treatment causes a more than 4.5 log10 reduction of virus titers.	(75)

EIDD-2801 exhibits excellent pharmacokinetic properties in cynomolgus monkeys and ferrets, and the single ascending dose oral pharmacokinetic (PK) profile in ferrets provides information on dose selection for clinical trials. Studies showed that EIDD-2801 achieves efficient biodistribution and anabolism *in vivo* at the orally administered concentration of 128 mg/kg and a single oral dose of EIDD-2801 at 128mg/kg in ferrets resulted in EIDD-1931 lung concentrations of 10.7 ± 1.2 nmol/g. And 7 days with twice-daily (b.i.d.) administration of 100 mg/kg concentration of EIDD-2801 were well tolerated by ferrets with no apparent adverse effects. By establishing a disease-relevant well-differentiated human airway epithelium model grown at air liquid interface (ALI), which simultaneously possesses the structural and cellular properties of human lung tissues, Toots et al. identified that the 50% cytotoxicity concentration (CC_50_) value of NHC for airway epithelial cells was 137 μM and the 50% effective concentration (EC_50_) value against IAV or IBV infection was approximately 0.06-0.08 μM. The specificity index (SI=CC_50_/EC_50_) against different influenza strains in human airway epithelial tissues was 1713-2283. Although IAV induced tissue damage could not be reversed in an epithelial model, drug treatment was able to significantly decrease released virus load in the supernatant (79). Similarly, Rosenke et al. detected comparable NHC/EIDD-1931 levels but not EIDD-2801 in the lung of all treated animals, as EIDD-2801 rapidly hydrolyzed to NHC (80).

Wahl et al. constructed a SARS-CoV-2 replication system in immunodeficient mice implanted with human lung tissue (LoM model). In depth analysis of LoM human lung tissue revealed that acute SARS-CoV-2 infection is highly pathogenic and induces a strong and sustained chemokine response of type I interferons and inflammatory cytokines. Therapeutic results indicated that molnupiravir/EIDD-2801 administration greatly reduced the number of infectious particles in LoM human lung tissue by more than 25,000 times (p=0.0002). When treatment was initiated 48 h post-exposure, the virus titer was significantly reduced by 96% (P=0.0019). It is worth noting that the earlier of molnupiravir treatment was initiated after SARS-CoV-2 exposure, the more pronounced in viral replication reduction. Results of prophylactic experiments showed that molnupiravir reduced the virus titer by more than 100,000-fold. In addition, a larger number of cell debris and nucleoprotein positive cells can be found in the alveoli of control mice compared with mice in the molnupiravir group, indicating that molnupiravir is highly effective in preventing SARS-CoV-2 infection and its pathogenic effect *in vivo* (50). Using a prophylactic and therapeutic dose escalation C57BL/6 mouse model, Sheahan et al. showed that prophylactic or therapeutic administration of EIDD-2801 significantly diminished or prevented body weight loss, greatly reduced lung hemorrhage and decreased virus titer in the lungs in a dose-dependent manner after SARS-CoV infection. Thus, prophylactic and therapeutic EIDD-2801 is a powerful antiviral agent capable of preventing SARS-CoV replication and disease. This study also found that the degree of clinical benefit relies on the treatment time after infection (49).

EIDD-2801 showed the same potent inhibition of SARS-CoV-2 replication in a Syrian hamster model, and analysis of lung tissue samples revealed that EIDD-2801 was able to significantly reduce viral RNA genome copy number and infectious virus levels. Immunohistochemistry (IHC) analysis showed that the viral antigen content of the vehicle control group was significantly higher than that of the treated group, and the average value of viral antigen signal in vehicle groups was 4.71 times more than that of drug prevention group and 3.68 times more than drug treatment group (80). Abdelnabi et al. compared the efficacy of molnupiravir against different SARS-CoV-2 strains including Wuhan strain, B.1.1.7 and B.1.351 variants in the Syrian hamster infection model. Molnupiravir treatment significantly reduced viral RNA copies and infectious virus titers in the lung regardless of the SARS-CoV-2 variants. In addition, cumulative histopathological lung scores were notably improved, and no or focal bronchopneumonia, perivascular inflammation as well as perivascular oedema were observed in animals receiving molnupiravir compared with the control group (19). As more variant strains emerge in the future, this compound may become an important tool in the fight against the COVID-19 pandemic.

Although several mouse and hamster models of SARS-CoV-2 infection have been established, there is no study supporting virus transmission between species. In contrast, the ferret model recapitulates the major features of human influenza virus infection, with the manifestation and spread of virus infection similar to asymptomatic transmission in the human population of young adults. Ferrets have been used to evaluate the transmission, infection and efficacy of drug treatment of SARS-CoV-2, providing strong support for the development of therapeutic interventions. Drug efficacy evaluation against influenza virus in ferrets showed that oral therapeutic of NHC reduced viral loads by several orders of magnitude. NHC treated animals relieved fever, airway epithelial histopathology, and inflammation compared to controls. During the assessment of virus spread to small airways, therapeutic application of NHC markedly reduced influenza virus titers in bronchioloalveolar lavage fluid and lung tissue sections. Immunohistochemical detection showed little virus infection in nasal turbinate epithelial layer and lung sections in EIDD-2801 treated animals, further demonstrating the effectiveness of the drug in inhibiting viral invasion (79). Moreover, another study identified that EIDD-2801 could dramatically decrease SARS-CoV-2 load in the upper respiratory tract and thoroughly block virus spread in ferrets. Studies in the ferret model infected with SARS-CoV-2 demonstrated that molnupiravir treatment did not cause any obvious adverse effects, and the white blood cell and platelet counts in the animals remained within the normal range (78). These studies demonstrated that preventive and therapeutic treatment of molnupiravir is able to inhibit virus transmission among ferrets to some extent.

Moreover, the combined antiviral effect of favipiravir and molnupiravir in a SARS-CoV-2 Syrian hamster infection model was investigated. The results showed that suboptimal doses of favipiravir or molnupiravir lead to approximately 1.2 log10 reduction in lung infectious virus titers, the combination treatment causes a more than 4.5 log10 reduction of virus titers. Moreover, they found that the combined molnupiravir/favipiravir treatment impairs viral spread to contact hamsters. Mechanistic study revealed that the combination treatment increased viral genome mutation rate compared to the monotherapy treatment (75). In addition, several studies have demonstrated prophylactic and therapeutic efficacy of the drug against influenza A and B virus infections in disease-relevant human airway epithelial models, mouse models, and ferret models (47, 81, 82). The above studies provide strong evidence for the antiviral efficacy of molnupiravir.

Rapid bioanalytical methods for the quantification of NHC in human plasma and intracellular NHC-triphosphate (NHCtp) in peripheral blood mononuclear cells (PBMC) lysates were developed and validated by Parsons et al. The results showed that NHC was detected in plasma with a lower limit of quantification (LLOQ) of 1 ng/mL and the primary linearity is 1-5000 ng/mL. The LLOQ of NHCtp in PBMC lysates was 1 pmol/sample, with a calibration range of 1-1500 pmol/sample (83). Determination of molnupiravir in vital tissues and its bioactive form NHC in plasma, and NHCtp inside cells is helpful in determining the therapeutic efficacy of drugs and characterizing their pharmacokinetic properties, laying a good foundation for clinical study. Although studies have demonstrated good tolerance of multiple doses administration of molnupiravir/EIDD-2801 in different animals, the possible effects of this drug on fetal health are currently unknown. Thus, experimental studies remain to be conducted to evaluate possible adverse effects on fetal health before using this compound for treatment during pregnancy (47, 81).

## Antiviral Resistance

SARS-CoV-2 is a single-strand positive-sense RNA virus with a genome generally characterized by high error rates, high viral yields, short replication time, and abundant homologous and nonhomologous recombination (84). Therefore, ‘viral swarms’ consisting of different genomic mutant populations with different degrees of fitness are generated, which can rapidly develop drug resistance while maintaining overall viral fitness, posing a great challenge to the development of broad-spectrum antiviral drugs (54).

Currently, few studies are focusing on the resistance of SARS-CoV-2 variants to molnupiravir. Available studies on CoVs showed that molnupiravir solves the issue of CoVs resistance while ensuring safety and efficacy. Moreover, molnupiravir establishes a high genetic barrier to prevent viruses escaping from inhibition (49, 55). NHC is the metabolite of molnupiravir, which incorporates into viral RNA and replaces nucleoside cytidine, increasing the rate of viral mutations (85). However, after repeatedly exposing the influenza virus to the drug, according to genetic sequence analyses, none of these mutations became allele-dominant or mediated a strong resistance (85, 86). And the antiviral activity of NHC was not restricted by the natural amino acid variation of RdRp. Sheahan et al. found that molnupiravir had high antiviral potency against CoVs bearing resistance mutations to remdesivir (49). Agostini et al. passaged two lineages of MERS-CoV for 30 times in the presence of increasing NHC concentrations and tested the sensitivity to NHC, the results showed that little acquired resistance was generated (55). Conversely, the viral error catastrophe invoked by this compound may block resistance (85), as molnupiravir constructs a highly resistant genetic barrier capable of fighting influenza virus escape inhibition (47, 79, 87). However, adaptation through serial passaging *in vivo* may be required to better predict whether antiviral resistance to EIDD-2801/NHC could ultimately emerge (86). Since the loss of the nsp14 could increase the sensitivity of SARS-CoV to remdesivir, it is also necessary to investigate the effect of the proofreading activity of nsp14 on the antiviral activity of NHC (49). Study has demonstrated that the combined treatment of molnupiravir and favipiravir exhibit pronounced combined antiviral potency to SARS-CoV-2 in a hamster infection model. The combined medication could greatly reduce concerns about the development of resistance to molnupiravir (75). Moreover, it was reported that molnupiravir could significantly inhibit different SARS-CoV-2 variants in the Syrian hamster infection model (19).

## Clinical Studies

There are seven clinical trials for molnupiravir (https://clinicaltrials.gov/ct2/results?cond=COVID-19&term=molnupiravir), two of which are completed, three are recruiting, one is active but not recruiting, and one is terminated (**Table 4**).

**Table 4:** Clinical studies registered on ClinicalTrials.gov of molnupiravir for COVID-19.

Trial identifier	Study design	Start date	Target number of participants	Severity of COVID-19	Status	Intervention/treatment	Results	Side effects	Source/reference
NCT04392219	Allocation: Randomized; Intervention Model: Parallel Assignment; Masking: Double (Participant, Investigator); Primary Purpose: Treatment	10 April, 2020	130	NA	Completed on 19 July, 2021	Program I: a) 50 to 1600 mg EIDD-2801 or PBO powder-in bottle b) two single dose 100 mg EIDD-2801 in an open-label manner	Molnupiravir is well tolerated and has excellent dose-proportional pharmacokinetics with relatively low variability.	37.5% and 44.6% of subjects reported an adverse event in the single and multiple ascending doses group, respectively. Both groups had no apparent dose-related adverse event trends, with a higher incidence of adverse events in placebo group than molnupiravir group, and 93.3% of adverse events were mild.	https://www.clinicaltrials.gov/ct2/show/record/NCT04392219 https://www.clinicaltrials.gov/ProvidedDocs/19/NCT04392219/Prot_000.pdf (51, 88)
						Program II: a) 50 to 1600 mg EIDD-2801 or PBO powder-in bottle (64) b) two single dose 200 mg EIDD-2801 c) 50-800 mg EIDD-2801 or PBO capsules in an open-label manner twice daily (BID) for 5.5 days			
						Program III: a) 50-1600 mg EIDD-2801 or PBO powder-in bottle b) two single dose 200 mg EIDD-2801 in an open-label manner c) 50-800 mg EIDD-2801 or PBO capsules BID for 5.5 days			
NCT04405570	Allocation: Randomized Intervention Model: Parallel Assignment Masking: Double (Participant, Investigator) Primary Purpose: Treatment	16 June, 2020	204	Mild or moderate	Completed on 21 February, 2021	a) 200 mg EIDD-2801 or PBO for 5 days b) 400/800 mg EIDD-2801 or PBO BID for 5 days	Infectious virus isolation from participants administered 800 mg molnupiravir were highly reduced compared to participants administered placebo on Day 3. No virus isolated from participants received 400 or 800 mg molnupiravir compared with administrated placebo, on Day 5 after molnupiravir treatment. Molnupiravir reduced the ability of SARS-cov-2 to replicate in patients with early COVID-19 infection significantly.	The number of adverse events in this study was low, with twice-daily 800 mg group having the lowest incidence. 4 serious adverse events occurred and resulted in hospitalization not related with molnupiravir treatment.	(88, 89) https://clinicaltrials.gov/ct2/show/record/NCT04405570 https://www.businesswire.com/news/home/20210305005610/en/
NCT04575597	Allocation: Randomized Intervention Model: Parallel Assignment Masking: Double (Participant, Investigator) Primary Purpose: Treatment	5 October, 2020	1850	Mild to moderate with 1 risk factor	Active, not recruiting	a) 200/400/800 mg molnupiravir in capsule or PBO every 12 hours (Q12H) for 5 days b) 800 mg Molnupiravir (dose to be selected) in capsule or PBO Q12H for 5 days	Determine the efficacy, and safety/tolerability, of molnupiravir (MK-4482) in adults who reside with a person infected with COVID-19. Molnupiravir reduced the risk of hospitalization or death in COVID-19 patients by about 50%.	By day 29 of the trial, no deaths had been reported in patients treated with molnupiravir, compared with eight deaths in patients treated with placebo. the incidence of adverse events was comparable in the molnupiravir and placebo groups (35% and 40%, respectively), and the incidence of adverse events due to the drug was comparable in the two groups (12% and 11%, respectively).	(88) https://www.clinicaltrials.gov/ct2/show/record/NCT04575597 https://www.businesswire.com/news/home/20211001005189/en
NCT04746183	Allocation: Randomized Intervention Model: Sequential Assignment Masking: Quadruple (Participant, Care Provider, Investigator, Outcomes Assessor) Primary Purpose: Treatment	3 July, 2020	600	Mild or moderate	recruiting	300/600/800 mg EIDD-2801 or PBO BID for 10 doses (5 or 6 days) 1500 mg Nitazoxanide or PBO BID for 7 days 50 mg VIR-7832 or PBO by intravenous (IV) infusion 500 mg VIR-7831 or PBO by IV infusion	Molnupiravir was well tolerated at 300, 600 and 800 mg doses with no serious or severe adverse events. 800 mg twice daily as the recommended phase II dose.	Adverse events affect 9/12 (4/4 for 300 and 600 mg, 1/4 for 800 mg) and 5/6 participants on molnupiravir and controls, respectively. All were mild and included flulike and upper respiratory symptoms, headache, myalgia, diarrhoea and nausea, which were also consistent with symptomatic COVID-19 disease.	https://www.clinicaltrials.gov/ct2/show/record/NCT04746183?term=Molnupiravir&draw=1&rank=7 (90)
NCT04405739	Allocation: Randomized Intervention Model: Parallel Assignment Masking: Double (Participant, Investigator) Primary Purpose: Treatment	6 June, 2020	96	Mild to moderate but hospitalized	recruiting	6 kinds of dose EIDD-2801 BID for 5 days	Efficacy and safety of EIDD-2801 on SARS-CoV-2 virus shedding in newly hospitalized adults with polymerase chain reaction (PCR)-confirmed COVID-19.	Assessing	https://www.clinicaltrials.gov/ct2/show/record/NCT04405739?term=NCT04405739&draw=1&rank=1 (88)
NCT04939428	Allocation: Randomized Intervention Model: Parallel Assignment Masking: Triple (Participant, Investigator, Outcomes Assessor) Primary Purpose: Prevention	11 August, 2021	1332	Non-COVID	recruiting	800 mg molnupiravir or PBO Q12H for 5 days	Evaluate the efficacy and safety of mk-4482 for the prevention of COVID-19 in adults residing with a person with COVID-19.	Assessing	https://clinicaltrials.gov/ct2/show/record/NCT04939428 (88)
NCT04575584	Allocation: Randomized Intervention Model: Parallel Assignment Masking: Double (Participant, Investigator) Primary Purpose: Treatment	19 October, 2020	304	Severe, Hospitalized	Terminated	200/400/800 mg molnupiravir or PBO Q12H for 5 days	Evaluate the efficacy, safety, and pharmacokinetics of mk-4482 in hospitalized adults with COVID-19.	Terminated	https://www.clinicaltrials.gov/ct2/show/record/NCT04575584 (88)

The currently completed clinical trial (NCT04392219) is a randomized, double-blind, placebo-controlled, first-in-human study designed to evaluate the safety, tolerability, and pharmacokinetics of EIDD-2801 following oral administration to healthy volunteers. The actual study completion date is August 11, 2021 (https://www.clinicaltrials.gov/ct2/show/NCT04392219). This trial involved 130 participants and was sponsored by Ridgeback Biotherapeutics, LP. Researchers evaluated the tolerability, safety, and pharmacokinetics of single and multiple ascending doses of molnupiravir. Suitable subjects were randomized divided in a 3:1 ratio to molnupiravir or placebo group. Each cohort included 8 participants, 6 of whom received molnupiravir and 2 received placebo. Subjects received single oral doses of 50 to 1600 mg molnupiravir or placebo in the single-dose evaluation portion, and subjects received 500 to 800 mg molnupiravir or placebo orally twice daily for 5.5 days in the multiple-dose ascending portion. The results illustrated that molnupiravir was absorbed and well-tolerated. Generally, 37.5% and 44.6% of subjects reported an adverse event in the single and multiple ascending doses group, respectively. Both groups had no apparent dose-related adverse event trends, with a higher incidence of adverse events in the placebo group than the molnupiravir group, and 93.3% of adverse events were mild. No clinically significant findings or dose-related trends in clinical laboratory, vital signs, and electrocardiogram data were observed (51). Therefore, orally administration of clinically relevant doses to healthy volunteers, molnupiravir is well tolerated and has excellent dose-proportional pharmacokinetics with relatively low variability.

Another clinical trial (NCT04405570), which has been completed, is a phase IIa randomized, double-blind, placebo-controlled trial to evaluate the antiviral efficacy of EIDD-2801 by measuring the time of infectious virus elimination from nasopharyngeal swabs in individuals with COVID-19 (https://clinicaltrials.gov/ct2/show/NCT04405570). Eligible participants included unvaccinated adult outpatients who were diagnosed with SARS-CoV-2 infection and symptoms onset within 7 days (89). Participants were randomly divided into 1:1 to 200 mg molnupiravir or placebo group, or 3:1 to molnupiravir (400 or 800 mg) or placebo group twice a day for 5 days. Among 202 treated subjects, researchers found that infectious virus isolation from participants administered 800 mg molnupiravir reduced dramatically compared to participants administered placebo on Day 3 (1.9% vs. 16.7%) (P=0.016). Infectious virus isolation also reduced on Day 5 after molnupiravir treatment, as no virus isolated from participants received 400 or 800 mg molnupiravir compared with 11.1% (6/54) of those administrated placebo (p=0.03). Safety analyses from this trial supported that molnupiravir was well tolerated with no increase in treatment- or dose-related serious adverse events compared with the placebo group (89). Meanwhile, according to a notice announced by MERCK, there were 4 serious adverse events occurred and resulted in hospitalization not related with molnupiravir treatment. At the same time, the trial’s lead investigator, Dr. William Fischer believed that the results of this study confirmed the ability of molnupiravir to reduce SARS-CoV-2 replication in early COVID-19 patients (https://www.businesswire.com/news/home/20210305005610/en/).

The clinical trial currently stopping recruiting is a phase 2/3, randomized, placebo-controlled, double-blind clinical study (NCT04575597). This trial estimated to enroll 1850 unvaccinated and non-hospitalized adults with mild or moderate COVID-19 and at least one symptom or underlying medical condition associated with an increased risk of severe COVID-19, and the safety and tolerability of molnupiravir will be evaluated by the percentage of participants who were hospitalized and/or died through day 29 (https://clinicaltrials.gov/show/NCT04575597). At the interim analysis, 7.3% of patients who received molnupiravir were hospitalized through day 29, compared with 14.1% of placebo-treated patients who were hospitalized or died. This suggests that molnupiravir reduced the risk of hospitalization or death in COVID-19 patients by about 50%. By day 29 of the trial, no deaths had been reported in patients treated with molnupiravir, compared with eight deaths in patients treated with placebo. Efficacy of molnupiravir was not affected by the duration of symptom onset or potential risk factors, and the incidence of adverse events was comparable in the molnupiravir and placebo groups (35% and 40%, respectively), as well as the incidence of adverse events due to the drug was comparable in the two groups (12% and 11%, respectively). All participants analysis showed that the percentage of participants who were hospitalized or died through day 29 was lower in the molnupiravir group than in the placebo group (6.8% vs. 9.7%). One death was reported in the molnupiravir group and 9 were reported in the placebo group through day 29. Adverse events were reported in 30.4% of the molnupiravir group and 33.0% of the placebo group (44).

The terminated clinical trial (NCT04575584), is a phase 2/3, randomized, placebo-controlled, double-blind clinical study to evaluate the efficacy, safety, and pharmacokinetics of MK-4482 in hospitalized adults with mild, moderate, or severe COVID-19. It was terminated for business reasons (https://clinicaltrials.gov/ct2/show/NCT04575584).

Based on a planned interim analysis of data from the Phase II/III trials, the decision has been made to proceed with the Phase 3 portion of the study in outpatients (MOVeOUT). Hospitalized patients were excluded (MOVeIN) because data suggested that molnupiravir is unlikely to show clinical benefit in hospitalized patients, who typically had a longer duration of symptoms prior to study entry (87).

There are three recruiting trials (NCT04746183, NCT04405739, NCT04939428). AGILE (early phase platform trial for COVID-19) in phase 1 and phase 2 was first posted on February 9, 2021, with the sponsor of the University of Liverpool (https://clinicaltrials.gov/ct2/show/NCT04746183). In this trial, researchers aimed to evaluate the safety and optimal dose of molnupiravir in 600 participants with early symptomatic infection (adult outpatients with PCR-confirmed SARS-CoV-2 infection within 5 days of symptom onset and with at least one well controlled symptom including cardiovascular disease, chronic lung disease, immune deficiency, diabetes, BMI≥30 or hypertension requiring medication). The phase Ib results showed that molnupiravir was well tolerated at 300, 600 and 800 mg doses with no serious or severe adverse events. And a dose of 800 mg two times daily for 5 days was recommended for phase II evaluation (90).

The safety of molnupiravir/EIDD-2801 and its effect on viral shedding of SARS-CoV-2 (END-COVID) is on phase 2, sponsored by Ridgeback Biotherapeutics, LP. This trial anticipates enrolling 96 adult participants already hospitalized and with symptoms associated with COVID-19, and is a multi-center, randomized, double-blind, placebo-controlled study to evaluate viral shedding of SARS-CoV-2 in newly hospitalized adults to assess the efficacy and safety of molnupiravir for the treatment of COVID-19 (https://clinicaltrials.gov/ct2/show/NCT04405739).

The study of molnupiravir/MK-4482 for prevention infection of COVID-19 in adults (MK-4482-013/MOVe-AHEAD) first posted on June 25, 2021, is a Phase 3 trial aimed to determine the efficacy, safety and tolerability of molnupiravir in adults who reside with a person infected with COVID-19 patient and without confirmed or suspected COVID-19 (https://clinicaltrials.gov/ct2/show/NCT04939428).

## Safety and Drug Tolerability

As a prodrug of nucleoside analogue, NHC exerts antiviral effects by causing lethal mutations during the synthesis of viral RNA. However, due to the common intermediate of ribose diphosphate shared by RNA and DNA precursors, NHC possesses the potential toxic risk to the host. The potential host mutagenic activity of NHC was verified by a gene selection system at a concentration of 3 µM in A549-hACE2 cells (91). The same results were obtained from the Ames experiment that molnupiravir harbors potentially genetic toxicity (46). The above studies indicate that molnupiravir carries a risk of carcinogenesis, which raises concerns about the ongoing side effects of molnupiravir. However, the aforementioned experimental results were obtained solely at the cellular level, and it is worth mentioning that in Zhou et al.’s investigation, even after 32 days of treatment with 3 µM rNHC, the mutagenesis effect was not as strong as that of 1 minute of UV exposure (91). More significantly, genotoxicity should not be explained only by the cellular level tests. Mutagenicity assay mandated by the U.S. FDA provided strong evidence that the Ames test for molnupiravir lacked genotoxicity association (https://www.fda.gov/regulatory-information/search-fda-guidance-documents/s2r1-genotoxicity-testing-and-data-interpretation-pharmaceuticals-intended-human-use). The results from both the Pig-A Mutagenicity Assay and the Big Blue^®^ (cII Locus) Transgenic rodent assay, powerful tools to evaluate the perniciousness of mutagenicity in humans, have confirmed that administration of molnupiravir at higher dosages and for longer periods than in the clinic did not result in an increased mutation rate in animals (92), which greatly facilitated the initiation of clinical studies on molnupiravir, and studies on the safety and tolerability of molnupiravir in non-hospitalized COVID-19 patients are underway (https://www.clinicaltrials.gov/ct2/show/NCT04575597). Phase 1 clinical data of molnupiriavir have been published, in which participants were grouped to receive either a single ascending dose (50 mg-1600 mg) or multiple ascending doses (twice-daily dose of 50 mg-800 mg) of molnupiravir to study its tolerability. In the single-ascending-dose group, the proportion of participants who reported adverse events after administration of placebo was higher than that of molnupiravir (43.8% vs. 35.4%). The same situation was found in the multiple-ascending-dose group (50.0% of placebo group vs. 42.9% of molnupiravir group). 93% of those who experienced adverse reactions to molnupiravir were mild. In addition, pharmacokinetic assay showed that molnupiravir was well absorbed and that at a dose of 200-800 mg drug concentration in plasma was sufficient to effectively prevent SARS-CoV-2 transmission in animal models (51). In the phase 2a study, molnupiravir possessed well-tolerance with similar incidence of side events reported in all groups (52). The current phase 3 trial also showed that the number of adverse events in the molnupiravir group was comparable to that in the placebo group (30.4% vs. 33.0%) (44), which promoted the authorization of molnupiravir by the U.K. MHRA and U.S. FDA. And the safety and toxicity data of molnupiravir/NHC are summarized in **Table 5**.

**Table 5:** Safety and toxicity data of molnupiravir/NHC.

Safety testing	Cell lines or animal models	Key findings	Reference
Cellular and animal assay	Huh7, HCV replicon cells, HepG2, or human PBM cells, Swiss mice (SWR/J)	NHC did not show toxicity in cells, but a modest reduction in rRNA levels when concentrations greater than 50 μM. NHC did not change mitDNA, mitRNA or lactic acid levels. All mice survived during the 6-day treatment and the 24-day monitoring period. The no-effect dose level in mice after 6 days i.p. was 33 mg/kg/day.	(67)
Cellular level assay	Vero E6 cells and macrophages	NHC did not have significant effects on cell viability after 48 h incubation and below 12 µM. NHC displayed a moderate cytotoxicity at 24 and 48 µM, but without cell detachment and cell rounding.	(68)
Animal assay	ICR mice	NHC showed good tolerability in mice after 7-day repeated dosing with up to 1000 mg/kg/day doses.	(69)
Cellular level assay	A549-hACE2 cells	3 µM NHC treated for 32 days displays host mutational activity in A549-hACE2 cells. However, it was not as strong as that of 1 minute of UV exposure.	(91)
Pig-a mutagenicity assay and Big Blue^®^ (cII Locus) transgenic rodent assay	rodent mutagenicity models	Administration of molnupiravir at higher dosages and for longer periods than in the clinic did not result in an increased mutation rate in animals.	(92)
first-in-human, phase 1	Human	Adverse events after administration of placebo were higher than that of molnupiravir both in two groups (single-ascending-dose group: 43.8% vs. 35.4%; multiple-ascending-dose group: 50.0% vs. 42.9%)	(51)
phase 2a study	Human	Molnupiravir possessed well-tolerance with similar incidence of side events reported in all groups.	(89)
phase 3 trial	Human	The interim analysis showed that 7.3% of patients treated with molnupiravir were hospitalized through day 29, while 14.1% of placebo-treated patients were hospitalized or died. All participants analysis showed that the percentage of participants who were hospitalized or died through day 29 was lower in the molnupiravir group than in the placebo group (6.8% vs. 9.7%). One death was reported in the molnupiravir group and 9 were reported in the placebo group through day 29. Adverse events were reported in 30.4% of the molnupiravir group and 33.0% of the placebo group.	(44)

The long-term impact of molnupiravir on humans is impossible to ascertain from current evidence, which may require a more than 10-year exploration. Considering the clinical safety of molnupiravir, its efficacy against SARS-COV-2, and the risk of severe disease associated with COVID-19, short-term administration of molnupiravir in non-hospitalized COVID-19 patients appears to be a viable clinical strategy to control the pandemic.

## Conclusion and Perspective

RNA viruses pose a great risk to global human health and are a major source of emerging and re-emerging infectious diseases (93). Woolhouse and Brierly reported a catalogue of 214 human infectious RNA viruses, linking these viruses with metadata affecting some characteristics of their epidemiology, including the date of the first report of human infection, transmission in the population, route of transmission and host range. They believe that climate change, land use and urbanization will lead to the discovery of more RNA viruses, and those with pandemic potential may appear (94, 95). In the twenty-first century, there were three zoonotic coronavirus diseases that seriously affected people’s health: the SARS-CoV in 2002/3, the persistent MERS-CoV in 2012, and the current SARS-CoV-2 global pandemic. The emergence of these viruses poses great challenges to scientists, requiring them to quickly understand the pathogenic factors, develop diagnostic reagents, analyze virus characteristics, trace the origins of the virus, and develop drugs and vaccines.

**Figure 4:**
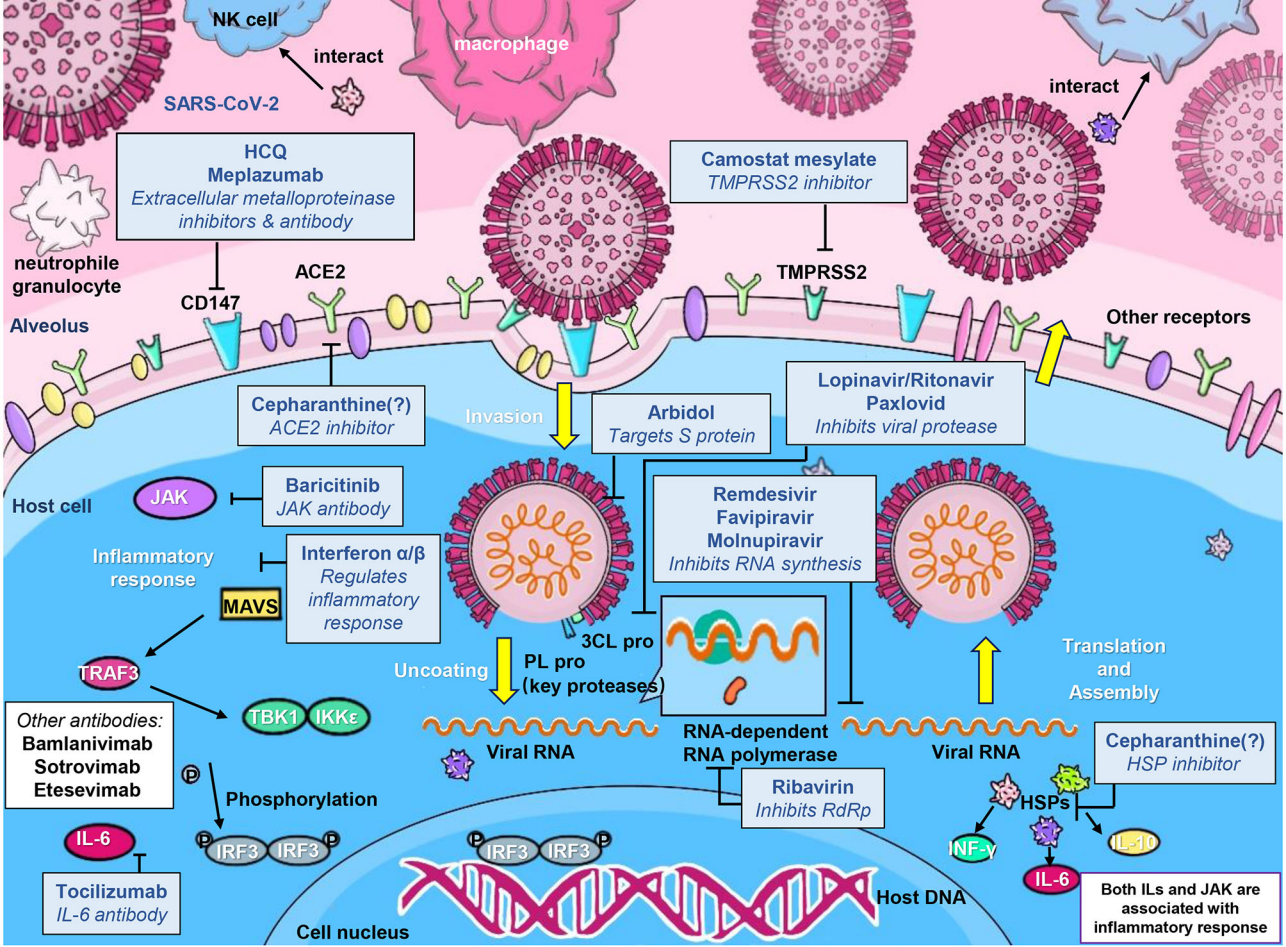
A schematic illustration of the mechanisms of developed anti-SARS-CoV-2 drugs. Anti-SARS-CoV-2 drugs can be classified into 4 main classes according to their different action mechanisms, class 1 drugs include Cepharanthine, HCQ, Arbidol, Camostat mesylate, and S protein targeted-antibodies including Bamlanivimab and Sotrovimab, which can prevent the entry of SARS-CoV-2; class 2 drugs include Ribavirin, Favipiravir, Remdesivir and Molnupiravir, which can inhibit the viral RNA synthesis; class 3 drugs include Lopinavir/Ritonavir and Paxlovid, which can inhibit viral 3CL protease; and class 4 drugs mainly include Tocilizumab and other inflammatory-alleviated drugs.

The global epidemic of COVID-19 has underlined the need for effective vaccines and antiviral drugs. Molnupiravir is a nucleoside analogue antiviral drug, which can be converted into prodrugs as needed. It has high oral bioavailability, broad-spectrum antiviral activity, and high barrier to developing antiviral resistance (96). These characteristics of molnupiravir contribute to its rapid development as COVID-19 therapeutics. Oral antiviral drugs play an important role in the treatment of patients with COVID-19, especially for lowering the risks of hospitalization and death from COVID-19. Oral antiviral drugs not only provide convenience for patients to use, but also reduce the pressure on healthcare medical systems. Moreover, their high stability does not require special storage and transportation methods, facilitating them to play a prominent role in the treatment of COVID-19 patients. On November 5, 2021, Pfizer announced that its oral antiviral drug paxlovid was safe and effective for SARS-CoV-2 infection. Compared with placebo, paxlovid reduced the risk of hospitalization or death of high-risk adult patients by 89% during the 28-day follow-up period. It blocks virus replication by inhibiting the activity of 3CL protease. On 12 December, the U.S. FDA approved the EUA of paxlovid for the treatment of mild-to-moderate adults and pediatric COVID-19 patients (https://www.fda.gov/news-events/press-announcements/coronavirus-covid-19-update-fda-authorizes-first-oral-antiviral-treatment-covid-19). In addition to molnupiravir and paxlovid, three more oral anti-COVID-19 drugs are in phase 3 clinical trials: the 3CL protease inhibitors s217622 (developed by Shionogi, Japan), the RdRp inhibitor AT-527 (jointly developed by Roche, Switzerland and Atea, USA), and the SARS-CoV-2 ACE2 and TMPRSS2 antagonist proxalutamide (initiated by Kintor Pharma, China) (14). Moreover, our group found that cepharanthine can effectively inhibit the entry and replication of GX_P2V, which has high homology with SARS-CoV-2, showing the potential antiviral activity of cepharanthine against SARS-CoV-2 (97, 98). Zhang et al. also showed that the combination therapy of cepharanthine and trifluoperazine has an extraordinary effect on the infection caused by B.1.351 variant (99). The discovery and application of more and more potent antiviral drugs will become a powerful weapon to block the COVID-19 epidemic. The comparative therapeutic information of molnupiravir, remdesivir, paxlovid and flavipiravir for COVID-19 are summarized in **Table 6**. And the action mechanisms of different anti-SARS-CoV-2 drugs were illustrated in **Figure 4**.

**Table 6:** Comparative information of molnupiravir, remdesivir, paxlovid and flavipiravir for COVID-19.

Drugs	Anti-viral Activity	Targets	Clinical Development Stage	Mechanism of Action	Reference
Flavipiravir	Favipiravir (T-705) can effectively inhibit RNA viruses such as influenza, Ebola, yellow fever, chikungunya, norovirus and enterovirus. It exhibited antiviral activity against SARS-CoV-2 in Vero E6 cells with EC_50_ values of 61.88 μM and CC_50_ values of 400 μM.	Nucleoside analogues in the form of adenine or guanine derivatives target the RNA-dependent RNA polymerase (RdRp) and block viral RNA synthesis.	At least 18 clinical trials registered in the Chinese Clinical Trial Registry (ChiCTR) and the International Clinical Trials Registry Platform (WHO ICTRP), proposing to use favipiravir in the treatment of COVID-19.	Favipiravir is an RdRp inhibitor. It converts to T-705-ribofuranosyl 5′-triphosphate by host enzymes and presumably acts as a nucleotide analog that selectively inhibits the viral RdRp or causes lethal mutagenesis upon incorporation into the virus RNA.	(100, 101)
Paxlovid	Paxlovid exhibited antiviral activity against SARS-CoV-2 (USA-WA1/2020 isolate) in differentiated normal human bronchial epithelial (dNHBE) cells with EC_50_ and EC_90_ values of 62 nM and 181 nM, respectively. Paxlovid had similar antiviral activity against SARS-CoV-2 variants include the Alpha, Beta, Gamma, Delta, and Lambda in cell culture.	Paxlovid targets the SARS-CoV-2 main protease (Mpro), referred to as 3C-like protease (3CLpro) or nsp5 protease.	Three clinical trials (NCT04962022, NCT04962230, and NCT04756531) of Paxlovid have been completed, five clinical trials (NCT04960202,NCT05064800, NCT05005312, NCT05032950, and NCT05047601) of Paxlovid are currently underway and will be disclosed shortly.	Paxlovid is a peptidomimetic inhibitor of the SARS-CoV-2 Mpro. Inhibition of SARS-CoV-2 Mpro renders it incapable of processing polyprotein precursors, preventing viral replication.	https://www.fda.gov/media/155050/download
Remdesivir	Remdesivir exhibited antiviral activity against SARS-CoV-2 in primary human airway epithelial (HAE) cells, Calu-3 and A549-hACE2 cell lines, with EC_50_ values of 9.9 nM, 280 nM and 115 nM, respectively. Compared to earlier lineage SARS-CoV-2 (lineage A) isolates, remdesivir retained similar antiviral activity (≤1.5-fold change) against clinical isolates of SARS-CoV-2 variants containing the P323L substitution in the viral polymerase including the Alpha, Beta, Delta, Gamma, and Epsilon variants.	Remdesivir triphosphate (RDVTP) acts as an analog of adenosine triphosphate (ATP) and competes with high selectivity (3.65-fold) over the natural ATP substrate for incorporation into nascent RNA chains. Targeting the SARS-CoV-2 RdRp, RDVTP results in delayed chain termination (position i+3) during replication of the viral RNA.	At least eight clinical studies on evaluating the safety and antiviral activity of remdesivir (GS-5734™) in participants with moderate COVID-19 have completed. And the results of four studies have been published.	Remdesivir is an inhibitor of the SARS-CoV-2 RdRp. As an adenosine nucleotide prodrug, remdesivir distributes into cells where it is metabolized to a nucleoside monophosphate intermediate. The nucleoside monophosphate is subsequently phosphorylated by cellular kinases to form the pharmacologically active nucleoside triphosphate metabolite (GS-443902). Remdesivir triphosphate (RDVTP) acts as an analog of adenosine triphosphate (ATP) and results in delayed chain termination during replication of the viral RNA.	https://www.fda.gov/media/137566/download
Molnupiravir	NHC is active against SARS-CoV-2 with EC_50_ values ranging between 0.67 to 2.66 µM in A549 cells and 0.32 to 2.03 µM in Vero E6 cells. NHC has similar activity against SARS-CoV-2 variants Alpha, Beta, Gamma, and Delta with EC_50_ values of 1.59, 1.77, 1.32 and 1.68 µM, respectively.	NHC 5’-triphosphate is used as a competitive alternative substrate and targets viral RdRp. It can integrate into viral RNA and lead to the accumulation of mutations in the viral genome, resulting in lethal mutations.	Clinical data supporting its EUA are based on data from 1,433 randomized subjects in the Phase 3 MOVe-OUT trial (NCT04575597).	Molnupiravir metabolizes to the cytidine nucleoside analogue NHC, which distributes into cells where NHC is phosphorylated to form the pharmacologically active ribonucleoside triphosphate (NHC-TP). NHC-monophosphate (NHC-MP) incorporation into SARS-CoV-2 RNA by the viral RNA polymerase (nsp12) results in an accumulation of mutations in the viral genome, leading to inhibition of viral replication.	https://www.fda.gov/media/155054/download

However, there are several points warrant emphasis. First, molnupiravir is not authorized for patients who are younger than 18 years old for it may affect bone and cartilage growth. In addition, molnupiravir is not recommended for use in pregnant individuals since it may cause fetal harm (https://www.fda.gov/news-events/press-announcements/coronavirus-covid-19-update-fda-authorizes-additional-oral-antiviral-treatment-covid-19-certain). According to MHRA, UK, patients who should not receive molnupiravir include those during breastfeeding as there is insufficient data. Since there is no study investigated the presence of molnupiravir or its metabolites in human milk and molnupiravir may have potential adverse reactions in the infant, breastfeeding is not recommended for the duration of molnupiravir treatment and for 4 days after the last dose (https://www.fda.gov/media/155054/download)(https://www.fda.gov/media/152929/download) (102). Its safety profile requires more research and needs to be strictly verified. Moreover, COVID-19 patients in a ‘highest’ risk group is one of the eligibility criteria, which include: a. Patients with active cancer; b. Patients with a hematological diseases and stem cell transplant recipients; c. Patients with renal disease; d. Patients with liver disease; e. Patients with immune-mediated inflammatory disorders; f. Primary immune deficiencies; g. HIV/AIDS; h. Solid organ transplant recipients; i. Rare neurological conditions; j. All patients with Down’s syndrome(www.gov.ukhttps://www.gov.uk/government/publications/regulatory-approval-of-lagevrio-molnupiravir/patient-information-leaflet-for-lagevrio). And no dosage adjustment is required in patients with renal or hepatic impairment since renal clearance and hepatic elimination are not a major route of NHC elimination (102). Second, molnupiravir should be administered as soon as possible after diagnosis of COVID-19 and within five days of symptom onset as it is less beneficial when initiated late. This requires efficient diagnostic test and issue remains about how early of asymptomatic individuals to start their treatment (88). Third, the efficacy of molnupiravir in patients with breakthrough infection after receiving vaccine still needs to be evaluated since more and more people get vaccinated worldwide. Fourth, will the widespread use of oral anti-COVID-19 drugs lead to the emergence of drug resistance? The recommended dose for molnupiravir is 800 mg (four 200 mg capsules) taken orally every 12 hours for five days, so it is unlikely to bring long-term pressure on the virus to induce drug-resistant variants. However, the generation of drug-resistant virus strains needs long-term and close monitoring. One solution to reduce drug resistance is “cocktail” therapy. The combined effect of molnupiravir and favipiravir has been studied and needs to be verified by more clinical trials (75). However, whether combination therapy will lead to more side effects is also a question that needs to be considered.

Recently, a newly emerging SARS-CoV-2 variant B.1.1.529 (Omicron) with 32 different mutation sites on the spike protein is attracting widespread attention. It was listed as a new variant of concern (VOC) by WHO on November 26, 2021 (https://www.who.int/en/activities/tracking-SARS-CoV-2-variants/). Several studies have investigated the immune escape ability of Omicron and showed that it extensively escapes to existing neutralizing antibodies, SARS-CoV-2 vaccines and convalescent sera (103–108). Whether this variant enhances the infectivity of SARS-CoV-2 and its impact on existing diagnosis, vaccines and therapies still need further study. Because molnupiravir’s mechanism of action is independent of mutations in the spike protein, it is expected to work against Omicron and other emerging variants, theoretically (102). *In vitro* study also indicates that molnupiravir retain their activity against the VOCs of Alpha, Beta, Gamma, Delta, and Omicron (109). SARS-CoV-2 Omicron variant is highly sensitive to molnupiravir. There is an urgent need for more clinical studies to evaluate the effectiveness of molnupiravir for treating Omicron infection (110). The effect of molnupiravir in breakthrough infections is unknown. What’s more, the role of molnupiravir in reducing hospitalization or death is expected to be limited among fully vaccinated individuals and people who had positive nucleocapsid antibody (20% at baseline) of recent or past SARS-CoV-2 infection (102). Currently, molnupiravir is not authorized for pre-exposure or post-exposure prophylaxis for prevention of COVID-19. Early termination of COVID-19 is a process that requires multiple collaborations including diagnostic tests, neutralizing antibodies, oral antiviral drugs, vaccines and public health measures. We hope that the development of scientific technology will bring us more and more tools to fight against the virus. And we look forward that we can effectively and comprehensively use these tools to end the epidemic as soon as possible.

## Author Contributions

JF, HF, YT, and LS designed the research. LT, ZP, ML, and FL read and analyzed the papers. XA and SZ participated in the discussion. JF, HF, LT, ZP, ML, and FL wrote and revised the manuscript. All authors contributed to the article and approved the submitted version.

## Funding

This study was supported by National Key Research and Development Program of China (grant No. BWS21J025, 20SWAQK22, 2020YFA0712102), Fundamental Research Funds for Central Universities (grant No. BUCTZY2022), H&H Global Research and Technology Center (grant No. H2021028), and National Natural Science Foundation of China (grant No. 82151224).

## Conflict of Interest

The authors declare that the research was conducted in the absence of any commercial or financial relationships that could be construed as a potential conflict of interest.

## Publisher’s Note

All claims expressed in this article are solely those of the authors and do not necessarily represent those of their affiliated organizations, or those of the publisher, the editors and the reviewers. Any product that may be evaluated in this article, or claim that may be made by its manufacturer, is not guaranteed or endorsed by the publisher.

## References

[1] Sun L Shen L Fan J Gu F Hu M An Y. Clinical Features of Patients With Coronavirus Disease 2019 From a Designated Hospital in Beijing, China. J Med Virol (2020) 92(10):2055–66. doi: 10.1002/jmv.25966 PMC7267635 32369208

[2] Sanders JM Monogue ML Jodlowski TZ Cutrell JB. Pharmacologic Treatments for Coronavirus Disease 2019 (COVID-19): A Review. JAMA (2020) 323(18):1824–36. doi: 10.1001/jama.2020.6019 32282022

[3] Hu Y Liu M Qin H Lin H An X Shi Z. Artemether, Artesunate, Arteannuin B, Echinatin, Licochalcone B and Andrographolide Effectively Inhibit SARS-CoV-2 and Related Viruses In Vitro. Front Cell Infect Microbiol (2021) 11:680127. doi: 10.3389/fcimb.2021.680127 34527599 PMC8435859

[4] Borba M Val F Sampaio VS Alexandre M Melo GC Brito M. Effect of High vs Low Doses of Chloroquine Diphosphate as Adjunctive Therapy for Patients Hospitalized With Severe Acute Respiratory Syndrome Coronavirus 2 (SARS-CoV-2) Infection: A Randomized Clinical Trial. JAMA Netw Open (2020) 3(4):e208857. doi: 10.1001/jamanetworkopen.2020.8857 32330277 PMC12124691

[5] Mitjà O Corbacho-Monné M Ubals M Alemany A Suñer C Tebé C. A Cluster-Randomized Trial of Hydroxychloroquine for Prevention of Covid-19. N Engl J Med (2021) 384(5):417–27. doi: 10.1056/NEJMoa2021801 PMC7722693 33289973

[6] Sakr Y Bensasi H Taha A Bauer M Ismail K. Camostat Mesylate Therapy in Critically Ill Patients With COVID-19 Pneumonia. Intensive Care Med (2021) 47(6):707–9. doi: 10.1007/s00134-021-06395-1 PMC8041240 33846824

[7] Zoufaly A Poglitsch M Aberle JH Hoepler W Seitz T Traugott M. Human Recombinant Soluble ACE2 in Severe COVID-19. Lancet Respir Med (2020) 8(11):1154–8. doi: 10.1016/S2213-2600(20)30418-5 PMC7515587 33131609

[8] Chen P Nirula A Heller B Gottlieb RL Boscia J Morris J. SARS-CoV-2 Neutralizing Antibody LY-CoV555 in Outpatients With Covid-19. N Engl J Med (2021) 384(3):229–37. doi: 10.1056/NEJMoa2029849 PMC7646625 33113295

[9] Xiang Y Nambulli S Xiao Z Liu H Sang Z Duprex WP. Versatile and Multivalent Nanobodies Efficiently Neutralize SARS-CoV-2. Science (2020) 370(6523):1479–84. doi: 10.1126/science.abe4747 PMC7857400 33154108

[10] Simonovich VA Burgos Pratx LD Scibona P Beruto MV Vallone MG Vázquez C. A Randomized Trial of Convalescent Plasma in Covid-19 Severe Pneumonia. N Engl J Med (2021) 384(7):619–29. doi: 10.1056/NEJMoa2031304 PMC7722692 33232588

[11] Zhu Z Lu Z Xu T Chen C Yang G Zha T. Arbidol Monotherapy is Superior to Lopinavir/Ritonavir in Treating COVID-19. J Infect (2020) 81(1):e21–3. doi: 10.1016/j.jinf.2020.03.060 PMC7195393 32283143

[12] Rocco P Silva PL Cruz FF Melo-Junior M Tierno P Moura MA. Early Use of Nitazoxanide in Mild COVID-19 Disease: Randomised, Placebo-Controlled Trial. Eur Respir J (2021) 58(1):2003725. doi: 10.1183/13993003.03725-2020 33361100 PMC7758778

[13] Hu Y Meng X Zhang F Xiang Y Wang J. The *In Vitro* Antiviral Activity of Lactoferrin Against Common Human Coronaviruses and SARS-CoV-2 is Mediated by Targeting the Heparan Sulfate Co-Receptor. Emerg Microbes Infect (2021) 10(1):317–30. doi: 10.1080/22221751.2021.1888660 PMC7919907 33560940

[14] Fan H Lou F Fan J Li M Tong Y. The Emergence of Powerful Oral Anti-COVID-19 Drugs in the Post-Vaccine Era. Lancet Microbe (2022) 3(2):e91. doi: 10.1016/S2666-5247(21)00278-0 34849495 PMC8616564

[15] Fan H Hong B Luo Y Peng Q Wang L Jin X. The Effect of Whey Protein on Viral Infection and Replication of SARS-CoV-2 and Pangolin Coronavirus *In Vitro* . Signal Transduct Target Ther (2020) 5(1):275. doi: 10.1038/s41392-020-00408-z 33235188 PMC7683587

[16] Wang Y Zhang D Du G Du R Zhao J Jin Y. Remdesivir in Adults With Severe COVID-19: A Randomised, Double-Blind, Placebo-Controlled, Multicentre Trial. Lancet (2020) 395(10236):1569–78. doi: 10.1016/S0140-6736(20)31022-9 PMC7190303 32423584

[17] Hung IF Lung KC Tso EY Liu R Chung TW Chu MY. Triple Combination of Interferon Beta-1b, Lopinavir-Ritonavir, and Ribavirin in the Treatment of Patients Admitted to Hospital With COVID-19: An Open-Label, Randomised, Phase 2 Trial. Lancet (2020) 395(10238):1695–704. doi: 10.1016/S0140-6736(20)31042-4 PMC7211500 32401715

[18] Solaymani-Dodaran M Ghanei M Bagheri M Qazvini A Vahedi E Hassan Saadat S. Safety and Efficacy of Favipiravir in Moderate to Severe SARS-CoV-2 Pneumonia. Int Immunopharmacol (2021) 95:107522. doi: 10.1016/j.intimp.2021.107522 33735712 PMC7951885

[19] Abdelnabi R Foo CS De Jonghe S Maes P Weynand B Neyts J. Molnupiravir Inhibits Replication of the Emerging SARS-CoV-2 Variants of Concern in a Hamster Infection Model. J Infect Dis (2021) 224(5):749–53. doi: 10.1093/infdis/jiab361 PMC8408768 34244768

[20] Good SS Westover J Jung KH Zhou XJ Moussa A La Colla P. AT-527, a Double Prodrug of a Guanosine Nucleotide Analog, Is a Potent Inhibitor of SARS-CoV-2 *In Vitro* and a Promising Oral Antiviral for Treatment of COVID-19. Antimicrob Agents Chemother (2021) 65(4):e02479–20. doi: 10.1128/AAC.02479-20 PMC8097421 33558299

[21] Cao B Wang Y Wen D Liu W Wang J Fan G. A Trial of Lopinavir-Ritonavir in Adults Hospitalized With Severe Covid-19. N Engl J Med (2020) 382(19):1787–99. doi: 10.1056/NEJMoa2001282 PMC7121492 32187464

[22] Vandyck K Deval J. Considerations for the Discovery and Development of 3-Chymotrypsin-Like Cysteine Protease Inhibitors Targeting SARS-CoV-2 Infection. Curr Opin Virol (2021) 49:36–40. doi: 10.1016/j.coviro.2021.04.006 34029993 PMC8075814

[23] Boras B Jones RM Anson BJ Arenson D Aschenbrenner L Bakowski MA. Preclinical Characterization of an Intravenous Coronavirus 3CL Protease Inhibitor for the Potential Treatment of COVID19. Nat Commun (2021) 12(1):6055. doi: 10.1038/s41467-021-26239-2 34663813 PMC8523698

[24] Ma C Sacco MD Hurst B Townsend JA Hu Y Szeto T. Boceprevir, GC-376, and Calpain Inhibitors II, XII Inhibit SARS-CoV-2 Viral Replication by Targeting the Viral Main Protease. Cell Res (2020) 30(8):678–92. doi: 10.1038/s41422-020-0356-z PMC7294525 32541865

[25] Cox RM Wolf JD Lieber CM Sourimant J Lin MJ Babusis D. Oral Prodrug of Remdesivir Parent GS-441524 is Efficacious Against SARS-CoV-2 in Ferrets. Nat Commun (2021) 12(1):6415. doi: 10.1038/s41467-021-26760-4 34741049 PMC8571282

[26] White KM Rosales R Yildiz S Kehrer T Miorin L Moreno E. Plitidepsin has Potent Preclinical Efficacy Against SARS-CoV-2 by Targeting the Host Protein Eef1a. Science (2021) 371(6532):926–31. doi: 10.1126/science.abf4058 PMC7963220 33495306

[27] Lenze EJ Mattar C Zorumski CF Stevens A Schweiger J Nicol GE. Fluvoxamine vs Placebo and Clinical Deterioration in Outpatients With Symptomatic COVID-19: A Randomized Clinical Trial. JAMA (2020) 324(22):2292–300. doi: 10.1001/jama.2020.22760 PMC7662481 33180097

[28] López-Medina E López P Hurtado IC Dávalos DM Ramirez O Martínez E. Effect of Ivermectin on Time to Resolution of Symptoms Among Adults With Mild COVID-19: A Randomized Clinical Trial. JAMA (2021) 325(14):1426–35. doi: 10.1001/jama.2021.3071 PMC7934083 33662102

[29] Horby P Lim WS Emberson JR Mafham M Bell JL Linsell L. Dexamethasone in Hospitalized Patients With Covid-19. N Engl J Med (2021) 384(8):693–704. doi: 10.1056/NEJMoa2021436 32678530 PMC7383595

[30] Salama C Han J Yau L Reiss WG Kramer B Neidhart JD. Tocilizumab in Patients Hospitalized With Covid-19 Pneumonia. N Engl J Med (2021) 384(1):20–30. doi: 10.1056/NEJMoa2030340 33332779 PMC7781101

[31] Lescure FX Honda H Fowler RA Lazar JS Shi G Wung P. Sarilumab in Patients Admitted to Hospital With Severe or Critical COVID-19: A Randomised, Double-Blind, Placebo-Controlled, Phase 3 Trial. Lancet Respir Med (2021) 9(5):522–32. doi: 10.1016/S2213-2600(21)00099-0 PMC8078879 33676590

[32] Pang J Xu F Aondio G Li Y Fumagalli A Lu M. Efficacy and Tolerability of Bevacizumab in Patients With Severe Covid-19. Nat Commun (2021) 12(1):814. doi: 10.1038/s41467-021-21085-8 33547300 PMC7864918

[33] Diurno F Numis FG Porta G Cirillo F Maddaluno S Ragozzino A. Eculizumab Treatment in Patients With COVID-19: Preliminary Results From Real Life ASL Napoli 2 Nord Experience. Eur Rev Med Pharmacol Sci (2020) 24(7):4040–7. doi: 10.26355/eurrev_202004_20875 32329881

[34] Davoudi-Monfared E Rahmani H Khalili H Hajiabdolbaghi M Salehi M Abbasian L. A Randomized Clinical Trial of the Efficacy and Safety of Interferon β-1a in Treatment of Severe COVID-19. Antimicrob Agents Chemother (2020) 64(9):e01061–20. doi: 10.1128/AAC.01061-20 PMC7449227 32661006

[35] Zhou Q Chen V Shannon CP Wei XS Xiang X Wang X. Interferon-α2b Treatment for COVID-19. Front Immunol (2020) 11:1061. doi: 10.3389/fimmu.2020.01061 32574262 PMC7242746

[36] Nhean S Varela ME Nguyen YN Juarez A Huynh T Udeh D. COVID-19: A Review of Potential Treatments (Corticosteroids, Remdesivir, Tocilizumab, Bamlanivimab/Etesevimab, and Casirivimab/Imdevimab) and Pharmacological Considerations. J Pharm Pract (2021) 0(0):8971900211048139. doi: 10.1177/08971900211048139 PMC10064180 34597525

[37] Malin JJ Suárez I Priesner V Fätkenheuer G Rybniker J. Remdesivir Against COVID-19 and Other Viral Diseases. Clin Microbiol Rev (2020) 34(1):e00162–20. doi: 10.1128/CMR.00162-20 PMC7566896 33055231

[38] Kim PS Read SW Fauci AS. Therapy for Early COVID-19: A Critical Need. JAMA (2020) 324(21):2149–50. doi: 10.1001/jama.2020.22813 33175121

[39] Hacisuleyman E Hale C Saito Y Blachere NE Bergh M Conlon EG. Vaccine Breakthrough Infections With SARS-CoV-2 Variants. N Engl J Med (2021) 384(23):2212–8. doi: 10.1056/NEJMoa2105000 PMC8117968 33882219

[40] Li M Lou F Fan H. SARS-CoV-2 Variants of Concern Delta: A Great Challenge to Prevention and Control of COVID-19. Signal Transduct Target Ther (2021) 6(1):349. doi: 10.1038/s41392-021-00767-1 34580279 PMC8475295

[41] Li M Lou F Fan H. SARS-CoV-2 Variants: A New Challenge to Convalescent Serum and mRNA Vaccine Neutralization Efficiency. Signal Transduct Target Ther (2021) 6(1):151. doi: 10.1038/s41392-021-00592-6 33839737 PMC8035603

[42] D’Amico F Rabaud C Peyrin-Biroulet L Danese S. SARS-CoV-2 Vaccination in IBD: More Pros Than Cons. Nat Rev Gastroenterol Hepatol (2021) 18(4):211–3. doi: 10.1038/s41575-021-00420-w PMC7816748 33473178

[43] Lou F Li M Pang Z Jiang L Guan L Tian L. Understanding the Secret of SARS-CoV-2 Variants of Concern/Interest and Immune Escape. Front Immunol (2021) 12:744242. doi: 10.3389/fimmu.2021.744242 34804024 PMC8602852

[44] Jayk Bernal A Gomes da Silva MM Musungaie DB Kovalchuk E Gonzalez A Delos Reyes V. Molnupiravir for Oral Treatment of Covid-19 in Nonhospitalized Patients. N Engl J Med (2021) 386(6):509–20. doi: 10.1056/NEJMoa2116044 PMC8693688 34914868

[45] Lee CC Hsieh CC Ko WC. Molnupiravir-A Novel Oral Anti-SARS-CoV-2 Agent. Antibiotics (Basel) (2021) 10(11):1294. doi: 10.3390/antibiotics10111294 34827232 PMC8614993

[46] Painter GR Natchus MG Cohen O Holman W Painter WP. Developing a Direct Acting, Orally Available Antiviral Agent in a Pandemic: The Evolution of Molnupiravir as a Potential Treatment for COVID-19. Curr Opin Virol (2021) 50:17–22. doi: 10.1016/j.coviro.2021.06.003 34271264 PMC8277160

[47] Toots M Yoon JJ Hart M Natchus MG Painter GR Plemper RK. Quantitative Efficacy Paradigms of the Influenza Clinical Drug Candidate EIDD-2801 in the Ferret Model. Transl Res (2020) 218:16–28. doi: 10.1016/j.trsl.2019.12.002 31945316 PMC7568909

[48] Yoon JJ Toots M Lee S Lee ME Ludeke B Luczo JM. Orally Efficacious Broad-Spectrum Ribonucleoside Analog Inhibitor of Influenza and Respiratory Syncytial Viruses. Antimicrob Agents Chemother (2018) 62(8):e00766–18. doi: 10.1128/AAC.00766-18 PMC6105843 29891600

[49] Sheahan TP Sims AC Zhou S Graham RL Pruijssers AJ Agostini ML. An Orally Bioavailable Broad-Spectrum Antiviral Inhibits SARS-CoV-2 in Human Airway Epithelial Cell Cultures and Multiple Coronaviruses in Mice. Sci Transl Med (2020) 12(541):eabb5883. doi: 10.1126/scitranslmed.abb5883 32253226 PMC7164393

[50] Wahl A Gralinski LE Johnson CE Yao W Kovarova M Dinnon KH 3rd. SARS-CoV-2 Infection is Effectively Treated and Prevented by EIDD-2801. Nature (2021) 591(7850):451–7. doi: 10.1038/s41586-021-03312-w PMC7979515 33561864

[51] Painter WP Holman W Bush JA Almazedi F Malik H Eraut N. Human Safety, Tolerability, and Pharmacokinetics of Molnupiravir, a Novel Broad-Spectrum Oral Antiviral Agent With Activity Against SARS-CoV-2. Antimicrob Agents Chemother (2021) 65(5):e02428–20. doi: 10.1128/AAC.02428-20 PMC8092915 33649113

[52] Fischer W Eron JJ Holman W Cohen MS Fang L Szewczyk LJ. Molnupiravir, an Oral Antiviral Treatment for COVID-19. medRxiv (2021) 14(628):eabl7430. doi: 10.1101/2021.06.17.21258639 PMC10763622 34941423

[53] Imran M Kumar Arora M Asdaq S Khan SA Alaqel SI Alshammari MK. Discovery, Development, and Patent Trends on Molnupiravir: A Prospective Oral Treatment for COVID-19. Molecules (2021) 26(19):5795. doi: 10.3390/molecules26195795 34641339 PMC8510125

[54] Pruijssers AJ Denison MR. Nucleoside Analogues for the Treatment of Coronavirus Infections. Curr Opin Virol (2019) 35:57–62. doi: 10.1016/j.coviro.2019.04.002 31125806 PMC7102703

[55] Agostini ML Pruijssers AJ Chappell JD Gribble J Lu X Andres EL. Small-Molecule Antiviral β-D-N (4)-Hydroxycytidine Inhibits a Proofreading-Intact Coronavirus With a High Genetic Barrier to Resistance. J Virol (2019) 93(24):e01348–19. doi: 10.1128/JVI.01348-19 PMC6880162 31578288

[56] Kabinger F Stiller C Schmitzová J Dienemann C Kokic G Hillen HS. Mechanism of Molnupiravir-Induced SARS-CoV-2 Mutagenesis. Nat Struct Mol Biol (2021) 28(9):740–6. doi: 10.1038/s41594-021-00651-0 PMC8437801 34381216

[57] Ronca SE Dineley KT Paessler S. Neurological Sequelae Resulting From Encephalitic Alphavirus Infection. Front Microbiol (2016) 7:959. doi: 10.3389/fmicb.2016.00959 27379085 PMC4913092

[58] Rivas F Diaz LA Cardenas VM Daza E Bruzon L Alcala A. Epidemic Venezuelan Equine Encephalitis in La Guajira, Colombia, 1995. J Infect Dis (1997) 175(4):828–32. doi: 10.1086/513978 9086137

[59] Carrera JP Forrester N Wang E Vittor AY Haddow AD López-Vergès S. Eastern Equine Encephalitis in Latin America. N Engl J Med (2013) 369(8):732–44. doi: 10.1056/NEJMoa1212628 PMC3839813 23964935

[60] Ehteshami M Tao S Zandi K Hsiao HM Jiang Y Hammond E. Characterization of β-D-N(4)-Hydroxycytidine as a Novel Inhibitor of Chikungunya Virus. Antimicrob Agents Chemother (2017) 61(4):e02395–16. doi: 10.1128/AAC.02395-16 PMC5365705 28137799

[61] Urakova N Kuznetsova V Crossman DK Sokratian A Guthrie DB Kolykhalov AA. β-D-N (4)-Hydroxycytidine Is a Potent Anti-Alphavirus Compound That Induces a High Level of Mutations in the Viral Genome. J Virol (2018) 92(3):e01965–17. doi: 10.1128/JVI.01965-17 PMC5774879 29167335

[62] Yamamoto T Koyama H Kurajoh M Shoji T Tsutsumi Z Moriwaki Y. Biochemistry of Uridine in Plasma. Clin Chim Acta (2011) 412(19-20):1712–24. doi: 10.1016/j.cca.2011.06.006 21689643

[63] Painter G Bluemling GR Natchus MG David G. N4-Hydroxycytidine and Derivatives and Anti-Viral Uses Related Thereto. U.S. Patent No 11147826B2 (2021).

[64] Steiner A Znidar D Ötvös SB Snead DR Dallinger D Kappe CO. A High-Yielding Synthesis of EIDD-2801 From Uridine. Eur J Org Chem (2020) 2020(43):6736–9. doi: 10.1002/ejoc.202001340 PMC7894511 33664631

[65] Vasudevan N Ahlqvist GP McGeough CP Paymode DJ Cardoso F Lucas T. A Concise Route to MK-4482 (EIDD-2801) From Cytidine. Chem Commun (Camb) (2020) 56(87):13363–4. doi: 10.1039/d0cc05944g 33030468

[66] Ahlqvist GP McGeough CP Senanayake C Armstrong JD Yadaw A Roy S. Progress Toward a Large-Scale Synthesis of Molnupiravir (MK-4482, EIDD-2801) From Cytidine. ACS Omega (2021) 6(15):10396–402. doi: 10.1021/acsomega.1c00772 PMC8153789 34056192

[67] Stuyver LJ Whitaker T McBrayer TR Hernandez-Santiago BI Lostia S Tharnish PM. Ribonucleoside Analogue That Blocks Replication of Bovine Viral Diarrhea and Hepatitis C Viruses in Culture. Antimicrob Agents Chemother (2003) 47(1):244–54. doi: 10.1128/AAC.47.1.244-254.2003 PMC149013 12499198

[68] Reynard O Nguyen XN Alazard-Dany N Barateau V Cimarelli A Volchkov VE. Identification of a New Ribonucleoside Inhibitor of Ebola Virus Replication. Viruses (2015) 7(12):6233–40. doi: 10.3390/v7122934 PMC4690858 26633464

[69] Painter GR Bowen RA Bluemling GR DeBergh J Edpuganti V Gruddanti PR. The Prophylactic and Therapeutic Activity of a Broadly Active Ribonucleoside Analog in a Murine Model of Intranasal Venezuelan Equine Encephalitis Virus Infection. Antiviral Res (2019) 171:104597. doi: 10.1016/j.antiviral.2019.104597 31494195

[70] Barnard DL Hubbard VD Burton J Smee DF Morrey JD Otto MJ. Inhibition of Severe Acute Respiratory Syndrome-Associated Coronavirus (SARSCoV) by Calpain Inhibitors and Beta-D-N4-Hydroxycytidine. Antivir Chem Chemother (2004) 15(1):15–22. doi: 10.1177/095632020401500102 15074711

[71] Pyrc K Bosch BJ Berkhout B Jebbink MF Dijkman R Rottier P. Inhibition of Human Coronavirus NL63 Infection at Early Stages of the Replication Cycle. Antimicrob Agents Chemother (2006) 50(6):2000–8. doi: 10.1128/AAC.01598-05 PMC1479111 16723558

[72] Gordon CJ Tchesnokov EP Schinazi RF Götte M. Molnupiravir Promotes SARS-CoV-2 Mutagenesis *via* the RNA Template. J Biol Chem (2021) 297(1):100770. doi: 10.1016/j.jbc.2021.100770 33989635 PMC8110631

[73] Menéndez-Arias L. Decoding Molnupiravir-Induced Mutagenesis in SARS-CoV-2. J Biol Chem (2021) 297(1):100867. doi: 10.1016/j.jbc.2021.100867 34118236 PMC8188802

[74] Kokic G Hillen HS Tegunov D Dienemann C Seitz F Schmitzova J. Mechanism of SARS-CoV-2 Polymerase Stalling by Remdesivir. Nat Commun (2021) 12(1):279. doi: 10.1038/s41467-020-20542-0 33436624 PMC7804290

[75] Abdelnabi R Foo CS Kaptein S Zhang X Do T Langendries L. The Combined Treatment of Molnupiravir and Favipiravir Results in a Potentiation of Antiviral Efficacy in a SARS-CoV-2 Hamster Infection Model. EBioMedicine (2021) 72:103595. doi: 10.1016/j.ebiom.2021.103595 34571361 PMC8461366

[76] Do T Donckers K Vangeel L Chatterjee AK Gallay PA Bobardt MD. A Robust SARS-CoV-2 Replication Model in Primary Human Epithelial Cells at the Air Liquid Interface to Assess Antiviral Agents. Antiviral Res (2021) 192:105122. doi: 10.1016/j.antiviral.2021.105122 34186107 PMC8233549

[77] Zhao J Guo S Yi D Li Q Ma L Zhang Y. A Cell-Based Assay to Discover Inhibitors of SARS-CoV-2 RNA Dependent RNA Polymerase. Antiviral Res (2021) 190:105078. doi: 10.1016/j.antiviral.2021.105078 33894278 PMC8059291

[78] Cox RM Wolf JD Plemper RK. Therapeutically Administered Ribonucleoside Analogue MK-4482/EIDD-2801 Blocks SARS-CoV-2 Transmission in Ferrets. Nat Microbiol (2021) 6(1):11–8. doi: 10.1038/s41564-020-00835-2 PMC7755744 33273742

[79] Toots M Yoon JJ Cox RM Hart M Sticher ZM Makhsous N. Characterization of Orally Efficacious Influenza Drug With High Resistance Barrier in Ferrets and Human Airway Epithelia. Sci Transl Med (2019) 11(515):eaax5866. doi: 10.1126/scitranslmed.aax5866 31645453 PMC6848974

[80] Rosenke K Hansen F Schwarz B Feldmann F Haddock E Rosenke R. Orally Delivered MK-4482 Inhibits SARS-CoV-2 Replication in the Syrian Hamster Model. Res Sq (2020). doi: 10.21203/rs.3.rs-86289/v1 PMC8052374 33863887

[81] Kumar D Trivedi N. Disease-Drug and Drug-Drug Interaction in COVID-19: Risk and Assessment. BioMed Pharmacother (2021) 139:111642. doi: 10.1016/j.biopha.2021.111642 33940506 PMC8078916

[82] Ribeiro IG Coelho-Dos-Reis J Fradico J Costa-Rocha I Silva LD Fonseca L. Remodeling of Immunological Biomarkers in Patients With Chronic Hepatitis C Treated With Direct-Acting Antiviral Therapy. Antiviral Res (2021) 190:105073. doi: 10.1016/j.antiviral.2021.105073 33887350

[83] Parsons TL Kryszak LA Marzinke MA. Development and Validation of Assays for the Quantification of β-D-N(4)-Hydroxycytidine in Human Plasma and β-D-N(4)-Hydroxycytidine-Triphosphate in Peripheral Blood Mononuclear Cell Lysates. J Chromatogr B Analyt Technol BioMed Life Sci (2021) 1182:122921. doi: 10.1016/j.jchromb.2021.122921 PMC8411588 34555541

[84] Perales C Domingo E. Antiviral Strategies Based on Lethal Mutagenesis and Error Threshold. Curr Top Microbiol Immunol (2016) 392:323–39. doi: 10.1007/82_2015_459 26294225

[85] Hampton T. New Flu Antiviral Candidate May Thwart Drug Resistance. JAMA (2020) 323(1):17. doi: 10.1001/jama.2019.20225 31910262

[86] Toots M Plemper RK. Next-Generation Direct-Acting Influenza Therapeutics. Transl Res (2020) 220:33–42. doi: 10.1016/j.trsl.2020.01.005 32088166 PMC7102518

[87] Şimşek-Yavuz S Komsuoğlu Çelikyurt FI. An Update of Anti-Viral Treatment of COVID-19. Turk J Med Sci (2021) 51(SI-1):3372–90. doi: 10.3906/sag-2106-250 PMC8771049 34391321

[88] Singh AK Singh A Singh R Misra A. Molnupiravir in COVID-19: A Systematic Review of Literature. Diabetes Metab Syndr (2021) 15(6):102329. doi: 10.1016/j.dsx.2021.102329 34742052 PMC8556684

[89] Fischer WA 2nd Eron JJ Jr Holman W Cohen MS Fang L Szewczyk LJ. A Phase 2a Clinical Trial of Molnupiravir in Patients With COVID-19 Shows Accelerated SARS-CoV-2 RNA Clearance and Elimination of Infectious Virus. Sci Transl Med (2022) 14(628):eabl7430. doi: 10.1126/scitranslmed.abl7430 34941423 PMC10763622

[90] Khoo SH Fitzgerald R Fletcher T Ewings S Jaki T Lyon R. Optimal Dose and Safety of Molnupiravir in Patients With Early SARS-CoV-2: A Phase I, Open-Label, Dose-Escalating, Randomized Controlled Study. J Antimicrob Chemother (2021) 76(12):3286–95. doi: 10.1093/jac/dkab318 PMC8598307 34450619

[91] Zhou S Hill CS Sarkar S Tse LV Woodburn B Schinazi RF. β-D-N4-Hydroxycytidine Inhibits SARS-CoV-2 Through Lethal Mutagenesis But Is Also Mutagenic To Mammalian Cells. J Infect Dis (2021) 224(3):415–9. doi: 10.1093/infdis/jiab247 PMC8136050 33961695

[92] Zeller A Pfuhler S Albertini S Bringezu F Czich A Dietz Y. A Critical Appraisal of the Sensitivity of *In Vivo* Genotoxicity Assays in Detecting Human Carcinogens. Mutagenesis (2018) 33(2):179–93. doi: 10.1093/mutage/gey005 29669112

[93] Woolhouse ME Brierley L McCaffery C Lycett S. Assessing the Epidemic Potential of RNA and DNA Viruses. Emerg Infect Dis (2016) 22(12):2037–44. doi: 10.3201/eid2212.160123 PMC5189130 27869592

[94] Woolhouse M Brierley L. Epidemiological Characteristics of Human-Infective RNA Viruses. Sci Data (2018) 5:180017. doi: 10.1038/sdata.2018.17 29461515 PMC5819479

[95] Zhang F Chase-Topping M Guo CG van Bunnik B Brierley L Woolhouse M. Global Discovery of Human-Infective RNA Viruses: A Modelling Analysis. PloS Pathog (2020) 16(11):e1009079. doi: 10.1371/journal.ppat.1009079 33253277 PMC7728385

[96] Jordan PC Stevens SK Deval J. Nucleosides for the Treatment of Respiratory RNA Virus Infections. Antivir Chem Chemother (2018) 26:2040206618764483. doi: 10.1177/2040206618764483 29562753 PMC5890544

[97] Fan HH Wang LQ Liu WL An XP Liu ZD He XQ. Repurposing of Clinically Approved Drugs for Treatment of Coronavirus Disease 2019 in a 2019-Novel Coronavirus-Related Coronavirus Model. Chin Med J (Engl) (2020) 133(9):1051–6. doi: 10.1097/CM9.0000000000000797 PMC7147283 32149769

[98] Li S Liu W Chen Y Wang L An W An X. Transcriptome Analysis of Cepharanthine Against a SARS-CoV-2-Related Coronavirus. Brief Bioinform (2021) 22(2):1378–86. doi: 10.1093/bib/bbaa387 PMC7929461 33423067

[99] Zhang S Huang W Ren L Ju X Gong M Rao J. Comparison of Viral RNA-Host Protein Interactomes Across Pathogenic RNA Viruses Informs Rapid Antiviral Drug Discovery for SARS-CoV-2. Cell Res (2021) 32(1):1–15. doi: 10.1038/s41422-021-00581-y PMC8566969 34737357

[100] Ghasemnejad-Berenji M Pashapour S. Favipiravir and COVID-19: A Simplified Summary. Drug Res (Stuttg) (2021) 71(3):166–70. doi: 10.1055/a-1296-7935 PMC8043595 33176367

[101] Campos D Fulco UL de Oliveira C Oliveira J. SARS-CoV-2 Virus Infection: Targets and Antiviral Pharmacological Strategies. J Evid Based Med (2020) 13(4):255–60. doi: 10.1111/jebm.12414 PMC7675315 33058394

[102] Singh AK Singh A Singh R Misra A. An Updated Practical Guideline on Use of Molnupiravir and Comparison With Agents Having Emergency Use Authorization for Treatment of COVID-19. Diabetes Metab Syndr (2022) 16(2):102396. doi: 10.1016/j.dsx.2022.102396 35051686 PMC8755553

[103] Li M Lou F Fan H. SARS-CoV-2 Variant Omicron: Currently the Most Complete “Escapee” From Neutralization by Antibodies and Vaccines. Signal Transduct Target Ther (2022) 7(1):28. doi: 10.1038/s41392-022-00880-9 35091532 PMC8795721

[104] Cao Y Wang J Jian F Xiao T Song W Yisimayi A. Omicron Escapes the Majority of Existing SARS-CoV-2 Neutralizing Antibodies. Nature (2021). doi: 10.1038/s41586-021-04385-3 PMC8866119 35016194

[105] Planas D Saunders N Maes P Guivel-Benhassine F Planchais C Buchrieser J. Considerable Escape of SARS-CoV-2 Omicron to Antibody Neutralization. Nature (2021) 602(7898):671–5. doi: 10.1038/s41586-021-04389-z 35016199

[106] Liu L Iketani S Guo Y Chan JF Wang M Liu L. Striking Antibody Evasion Manifested by the Omicron Variant of SARS-CoV-2. Nature (2021) 602(7898):676–81. doi: 10.1038/s41586-021-04388-0 35016198

[107] Cele S Jackson L Khoury DS Khan K Moyo-Gwete T Tegally H. Omicron Extensively But Incompletely Escapes Pfizer BNT162b2 Neutralization. Nature (2021) 602(7898):654–6. doi: 10.1038/s41586-021-04387-1 PMC8866126 35016196

[108] Cameroni E Bowen JE Rosen LE Saliba C Zepeda SK Culap K. Broadly Neutralizing Antibodies Overcome SARS-CoV-2 Omicron Antigenic Shift. Nature (2021). doi: 10.1038/s41586-021-04386-2 PMC9531318 35016195

[109] Vangeel L Chiu W De Jonghe S Maes P Slechten B Raymenants J. Remdesivir, Molnupiravir and Nirmatrelvir Remain Active Against SARS-CoV-2 Omicron and Other Variants of Concern. Antiviral Res (2022) 198:105252. doi: 10.1016/j.antiviral.2022.105252 35085683 PMC8785409

[110] Li P Wang Y Lavrijsen M Lamers MM de Vries AC Rottier RJ. SARS-CoV-2 Omicron Variant is Highly Sensitive to Molnupiravir, Nirmatrelvir, and the Combination. Cell Res (2022) 71(3):166–70. doi: 10.1038/s41422-022-00618-w PMC8771185 35058606

